# Configuration Synthesis and Performance Analysis of 1T2R Decoupled Wheel-Legged Reconfigurable Mechanism

**DOI:** 10.3390/mi16080903

**Published:** 2025-07-31

**Authors:** Jingjing Shi, Ruiqin Li, Wenxiao Guo

**Affiliations:** School of Mechanical Engineering, North University of China, Taiyuan 030051, China; 18135306743@163.com

**Keywords:** configuration synthesis, decoupled parallel mechanism, reconfigurable, performance index, dimension optimization

## Abstract

A method for configuration synthesis of a reconfigurable decoupled parallel mechanical leg is proposed. In addition, a configuration evaluation index is proposed to evaluate the synthesized configurations and select the optimal one. Kinematic analysis and performance optimization of the selected mechanism’s configuration are carried out, and the motion mode of the robot’s reconfigurable mechanical leg is selected according to the task requirements. Then, the robot’s gait in walking mode is planned. Firstly, based on bionic principles, the motion characteristics of a mechanical leg based on a mammalian model and an insect model were analyzed. The input and output characteristics of the mechanism were analyzed to obtain the reconfiguration principle of the mechanism. Using type synthesis theory for the decoupled parallel mechanism, the configuration synthesis of the chain was carried out, and the constraint mode of the mechanical leg was determined according to the constraint property of the chain and the motion characteristics of the moving platform. Secondly, an evaluation index for the complexity of the reconfigurable mechanical leg structure was developed, and the synthesized mechanism was further analyzed and evaluated to select the mechanical leg’s configuration. Thirdly, the inverse position equations were established for the mechanical leg in the two motion modes, and its Jacobian matrix was derived. The degrees of freedom of the mechanism are completely decoupled in the two motion modes. Then, the workspace and motion/force transmission performance of the mechanical leg in the two motion modes were analyzed. Based on the weighted standard deviation of the motion/force transmission performance, the global performance fluctuation index of the mechanical leg motion/force transmission is defined, and the structural size parameters of the mechanical leg are optimized with the performance index as the optimization objective function. Finally, with the reconfigurable mechanical leg in the insect mode, the robot’s gait in the walking operation mode is planned according to the static stability criterion.

## 1. Introduction

Wheel-legged robots have the advantages of both wheeled robots and legged robots, meaning they can not only move quickly on flat terrain but also have the ability to overcome obstacles. Compared with the serial mechanical leg, the parallel mechanical leg has the advantages of structural stability and a high load capacity, but it entails significant motion coupling and its control is complex compared with the series mechanism. The performance requirements of mobile robots differ depending on the terrain and working environment. For example, in the face of complex terrain, the robot needs to be flexible and changeable and, in the face of heavy-duty tasks, it must have high load-bearing performance. A reconfigurable decoupled parallel mechanism can switch between different motion modes according to the requirements of the task and its control difficulty is low, which can meet the needs of the mobile robot leg.

Robots with both wheels and legs can be divided into two types: those in which the wheels and legs are combined and those in which they are independent of one another [1]. In robots with a wheel–leg combination, the wheel is directly installed at the end of the leg, and switching between the wheel and leg motion modes is realized through the motion control of the leg pairs and the locking and unlocking of the wheels [2,3,4,5]. Switching between the wheel and leg modes is relatively simple, which reduces the complexity of the control of the mechanical leg on different terrains. Tedeschi et al. [6] designed a six-wheel-legged robot named Cassino Hexapod III, which adopts a wheel–leg combination. Each leg of the robot has three revolute pairs, which enable the robot to cross obstacles under the premise of limiting the wheels’ movement. In the wheel mode of a robot in which the wheels and legs work independently, the legs are folded while the wheels are in contact with the ground, relying on the rolling of the wheels to realize rapid movement. In the leg mode, the wheels are separated from the ground, and the movement is realized by the coordinated movement of the joints [7,8,9]. Xu et al. [10] proposed a six-wheel-legged mobile robot called NOROS. Each leg has three driving pairs and adopts a combination of independent wheels and legs, where the wheels are mounted on the mechanical legs.

The input and output of the decoupled parallel mechanism maintain a one-to-one correspondence, which can significantly reduce the complexity of the control of the parallel mechanism, so it can perform more complex actions. Zeng et al. [10] proposed a 2CRR + RRRR (C denotes a cylindrical pair; R denotes a revolute pair) decoupled parallel mechanism and derived its Jacobian matrix via the inverse position solution. The results show that the Jacobian matrix of the mechanism is a diagonal matrix, which verifies the decoupled characteristics of the mechanism. Cao et al. [11] proposed a method for the type synthesis of a 1T3R decoupled parallel mechanism based on the kinematic characteristics of the mechanism and screw theory. Xu et al. [12] conducted configuration synthesis of a 2R1T parallel mechanism and a 2R parallel mechanism with two completely decoupled rotational degrees of freedom based on the relationship between the constrained screw and the rotation axis of the mechanism. Qu et al. [13] carried out the configuration synthesis of a 2R1T parallel mechanism with redundant constraints based on the redundant constraints of the parallel mechanism. Zhang et al. [14] established a model for mapping the input and output vectors of a 1T2R fully decoupled parallel mechanism based on screw theory and synthesized a series of 1T2R completely decoupled parallel mechanisms. Li et al. [15] proposed a method for configuration synthesis of a high-stiffness 3T completely decoupled parallel mechanism based on screw theory, constructing closed-loop units in the chains to enhance the stiffness of the parallel mechanism. Wang et al. [16] proposed a method for configuration synthesis of a 3T2R decoupled hybrid mechanism based on screw theory and the atlas method and put forward the decoupled conditions for 3T and 2R parallel mechanisms according to their degrees of freedom and decoupled characteristics.

The traditional parallel mechanism has certain degrees of freedom. When facing the requirements of a task on diversified terrain, the number of degrees of freedom required by the mechanism will change, which may increase the complexity and control difficulty of the mechanism. A reconfigurable parallel mechanism can improve the adaptability to the working environment by changing its configuration. Wang et al. [17] proposed an 8R metamorphic mechanism based on origami folding, which can switch between 2 and 5 degrees of freedom, and designed a quadruped mobile robot with this metamorphic mechanism as a reconfigurable torso. Hu et al. [18] proposed a lockable parallel spherical pair S_dm_ based on screw theory and constructed a 2-SdmPU/SPS parallel mechanism by combining the S_dm_ pair, a prismatic pair, and a spherical pair, which can realize multi-mode motion of fixed-axis rotation and a one-dimensional variable axis. Palpacelli et al. [19] proposed a lockable spherical pair *S_r_*. By locking the rotating shafts of *S_r_* in different directions, the mechanism can switch between three kinematic pair modes: a spherical pair, universal pair, and revolute pair. Ye et al. [20] proposed the 3SvPS reconfigurable parallel mechanism by combining the vA (variable-Axis) pair, the prismatic pair, and the spherical pair. When the vA pair is in different phases, the mechanism can switch between four motion modes: 3T3R, 2T3R, 1T3R, and 1T2R. Yuan et al. [21] proposed the TS pair based on the traditional universal pair; they then proposed a 3(TS)P(TS) reconfigurable mechanism based on the TS pair that can switch between 1T, 1T1R, 2T1R, and other motion modes by changing the phase of the motion axis of the TS pair. Inspired by single-vertex origami, Kuang et al. [22] proposed a 5R spherical mechanism and combined a reconfigurable kinematic pair with prismatic and spherical pairs to construct a 3-(5R)PS reconfigurable parallel mechanism.

Based on the functional requirements of a reconfigurable four-wheel-legged robot, this study proposes a method for configuration synthesis of a reconfigurable decoupled wheel-legged mechanical leg. In the second section, the relationship between the input and output of the mechanical leg and the motion characteristics of the horse and ant limb structures is analyzed, and the reconfiguration principle of the mechanical leg is obtained. In the third section, the configuration synthesis of the chain is carried out based on a lockable universal pair. According to the constraint properties of the chain and the motion characteristics of the moving platform, the mechanism’s constraint mode is determined and the chain type is extended. In the fourth section, an evaluation index of the structural complexity of the reconfigurable mechanical leg is proposed, and the synthesized mechanism is further analyzed and evaluated to determine the configuration of the mechanical leg. In the fifth section, the inverse position equations of the mechanical leg in the two motion modes are established; in addition, the Jacobian matrix of the mechanical leg is derived, and its decoupled motion characteristics are analyzed. In the sixth section, the workspace and motion/force transmission performance of the mechanical leg in the two motion modes are analyzed and compared. The global performance fluctuation index of motion/force transmission of the mechanical leg is defined based on the weighted standard deviation of motion/force transmission performance. The particle swarm optimization algorithm is used to optimize the structural size parameters of the mechanical leg. As shown in the seventh section, the insect mode is selected as the motion mode of the mechanical leg when the robot is in the walking operation mode. Finally, based on the stability criterion of the robot, the robot’s gait is planned.

## 2. Configuration Synthesis of the Reconfigurable Decoupled Mechanical Leg

When a mobile robot needs to complete heavy tasks, its legs need a high load capacity; meanwhile, when it faces complex terrain, its legs need to be capable of flexible movement. The mammalian configuration has the advantages of a high load capacity and good stability, but it has the disadvantages of a small support area and a high center of gravity. The advantage of the insect-type configuration is that the support area is large and flexible, while its disadvantage is that its load capacity is low [23]. The reconfigurable mechanical leg with two motion modes has the above advantages and can switch between different motion modes according to the task requirements. In this study, the horse and ant are selected as models for designing the bionic structure of the reconfigurable mechanical leg.

### 2.1. Analysis of Biological Limbs’ Motion Characteristics

**Figure 1:**
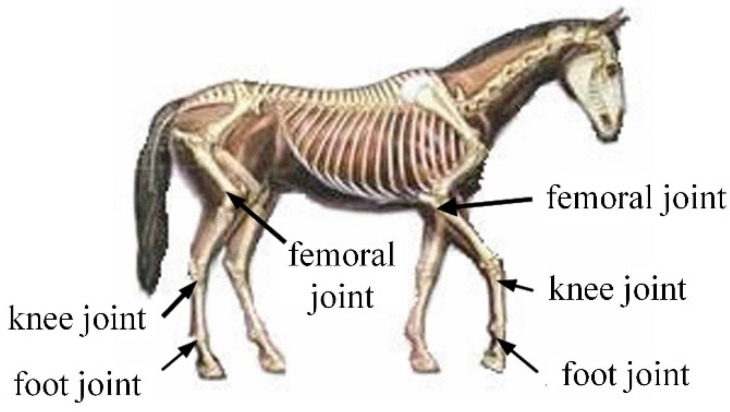
Skeleton and joint pairs of a horse.

Compared with other four-legged mammals, horses have a unique physiological structure and motion patterns. It has strong adaptability to environments with complex terrain and a high load-bearing capacity, and it was the main means of cargo transportation for thousands of years. Thus, the horse is chosen as the bionic research object for the mechanical leg in the mammalian mode, and its skeleton and joint pairs are analyzed and studied, as shown in Figure 1.

**Figure 2:**
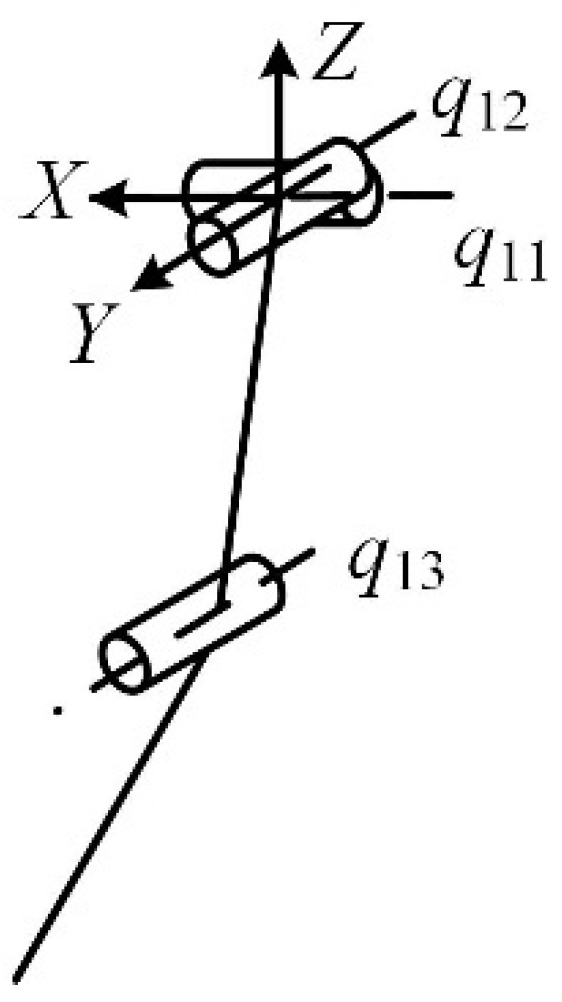
Mammalian one-legged configuration.

It can be seen in Figure 1 that the front and hind legs of the horse have three joints: the femoral, knee, and foot joints. The coordinate system is set at the femoral pair, as shown in Figure 2. The twist system of the mammalian one-legged configuration is

(1) $$\begin{array}{lllllllll} {\$}_{q11} & = & (1 & 0 & 0 & ; & 0 & 0 & 0)\\ {\$}_{q12} & = & (0 & 1 & 0 & ; & 0 & 0 & 0)\\ {\$}_{q13} & = & (0 & 1 & 0 & ; & m_{13} & 0 & n_{13}) \end{array}$$

where *m*_13_ and *n*_13_ are parameters related to the position of the kinematic pair of the mechanical leg.

The reciprocal wrench of the mammalian one-legged configuration is

(2) $$\begin{array}{lllllllll} {\$}_{q11}^{r} & = & (-n_{13} & 0 & m_{13} & ; & 0 & 0 & 0)\\ {\$}_{q12}^{r} & = & (0 & 1 & 0 & ; & 0 & 0 & 0)\\ {\$}_{q13}^{r} & = & (0 & 0 & 0 & ; & 0 & 0 & 1) \end{array}$$

As can be seen from Equation (2), the direction of the translational degree of freedom changes with the movement of the mechanical leg. The translation along the *Z*-axis enables the robot to adjust its leg length according to the height of the ground to adapt to a change in terrain. Considering that an unstructured terrain in a dangerous and complex environment requires the ability to cross obstacles, and given the technological conditions of the actual mechanical structure, rotation about the *X*-axis, rotation about the *Y*-axis, and translation along the *Z*-axis are selected as the degrees of freedom of the mammalian leg.

In the mammalian mode, the twist system of the mechanical leg is

(3) $$\begin{array}{l} {\$}_{11}=(\begin{array}{lllllll} 1 & 0 & 0 & ; & 0 & 0 & 0 \end{array})\\ {\$}_{12}=(\begin{array}{lllllll} 0 & 1 & 0 & ; & 0 & 0 & 0 \end{array})\\ {\$}_{13}=(\begin{array}{lllllll} 0 & 0 & 0 & ; & 0 & 0 & 1 \end{array}) \end{array}$$

**Figure 3:**
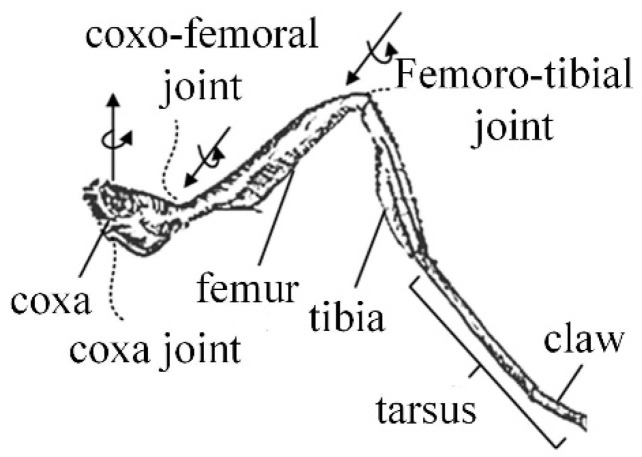
The leg structure of the ant.

Over a long period of evolution, insects have developed the ability to move flexibly over various complex terrains. As a common hexapod insect, ants can switch gaits according to the required walking speed and the terrain and have flexible motion performance. Therefore, ants are selected as the bionic research object for the mechanical leg in the insect mode. The ant’s body and leg structures are shown in Figure 3.

**Figure 4:**
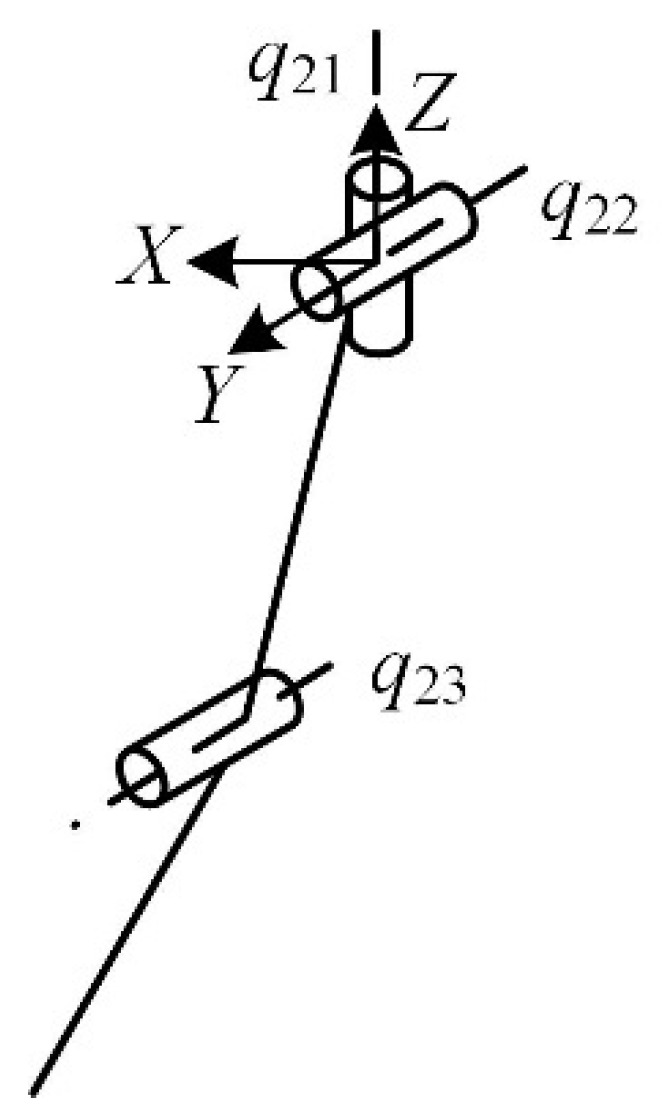
The insect-like single-leg configuration.

It can be seen in Figure 3 that the main joints of the ant leg are the coxa, coxa-femoral, and femoro-tibial joints. The configuration of the insect-type single leg is analyzed, as shown in Figure 4. The twist system of the insect-like leg structure is

(4) $$\begin{array}{lllllllll} {\$}_{q21} & = & (0 & 0 & 1 & ; & 0 & 0 & 0)\\ {\$}_{q22} & = & (0 & 1 & 0 & ; & 0 & 0 & 0)\\ {\$}_{q23} & = & (0 & 1 & 0 & ; & m_{23} & 0 & n_{23}) \end{array}$$

where *m*_23_ and *n*_23_ are parameters related to the position of the kinematic pair of the mechanical leg.

The reciprocal wrench of the insect one-legged configuration is

(5) $$\begin{array}{lllllllll} {\$}_{q21}^{r} & = & (-n_{23} & 0 & m_{23} & ; & 0 & 0 & 0)\\ {\$}_{q22}^{r} & = & (0 & 1 & 0 & ; & 0 & 0 & 0)\\ {\$}_{q23}^{r} & = & (0 & 0 & 0 & ; & 1 & 0 & 0) \end{array}$$

As can be seen from Equation (5), the direction of the translational degree of freedom changes with the movement of the mechanical leg. Considering that an unstructured terrain in a dangerous and complex environment requires the ability to cross obstacles, and given the technological conditions of the actual mechanical structure, rotation about the *Z*-axis, rotation about the *Y*-axis, and translation along the *Z*-axis are selected as the degrees of freedom of the insect legs.

In the insect mode, the twist system of the mechanical leg is

(6) $$\begin{array}{l} {\$}_{21}=(\begin{array}{lllllll} 0 & 0 & 1 & ; & 0 & 0 & 0 \end{array})\\ {\$}_{22}=(\begin{array}{lllllll} 0 & 1 & 0 & ; & 0 & 0 & 0 \end{array})\\ {\$}_{23}=(\begin{array}{lllllll} 0 & 0 & 0 & ; & 0 & 0 & 1 \end{array}) \end{array}$$

Equations (3) and (6) indicate that when the mechanical leg is in the mammalian mode, the degrees of freedom are R*_X_*R*_Y_*T*_Z_*; when the mechanical leg is in the insect mode, the degrees of freedom are R*_Z_*R*_Y_*T*_Z_*.

### 2.2. Input–Output Analysis of 1T2R Decoupled Parallel Mechanism

The instantaneous motion of the moving platform of the parallel mechanism can be expressed by the twist [14] of the mechanism’s chain, namely,

(7) $$V=\left[\begin{array}{c} v\\ \omega \end{array}\right]=\sum_{j=1}^{F_{i}}{\;\dot{q}}_{ij}{\$}_{ij}\;\;\;\left(i=1,2,\cdots ,n\right)$$

where ***V*** is the velocity vector of the moving platform; ***v*** denotes the linear velocity of the moving platform; ***ω*** represents the angular velocity of the moving platform; *F_i_* is the connectivity of the *i*-th chain; *n* is the number of chains of the mechanism;${\;\dot{q}}_{ij}$ represents the linear or angular velocity of the *j*-th kinematic pair in the *i*-th chain; and ***$****_ij_* is the twist of the *j*-th single-degree-of-freedom kinematic pair in the *i*-th chain.

The chain constraint wrench limits the translational degree of freedom of the moving platform parallel to the *X*- and *Y*-axes, such that any component parallel to the *X*- and *Y*-axes in ***v*** is always 0.

The transmission wrench screw ***$***_T_ [15] represents the generalized force transmitted by the driving pair to the moving platform, which has a zero reciprocity product with all other twists except for the actuated twist. Taking the reciprocal product of both sides of Equation (7) with ***$***_T*i*_ yields

(8) $${\$}_{Ti}\circ V={\;\dot{q}}_{ij}{\$}_{Ti}\circ {\$}_{ij}$$

The matrix form of Equation (8) is

(9) $$J_{\mathrm{dir}}V=J_{\mathrm{inv}}\dot{q}\;$$

where ***J***_dir_ is the mechanism’s output Jacobian matrix and ***J***_inv_ is the its input Jacobian matrix.

If ***J***_inv_ is invertible, Equation (9) can be written as

(10) $$\dot{q}=J_{\mathrm{inv}}^{-1}J_{\mathrm{dir}}V=JV$$

where ***J*** is the Jacobian matrix of the mechanism.

When ***J*** is a diagonal matrix, the mechanism is a completely decoupled parallel mechanism. It can be seen from Equation (10) that ***J***_inv_ is a diagonal matrix. According to the matrix operation rules, $J_{\mathrm{inv}}^{-1}$ is still a diagonal matrix. When ***J***_dir_ is a diagonal matrix, ***J*** is a diagonal matrix, and the mechanism is a completely decoupled parallel mechanism.

### 2.3. Reconfiguration Principle of Mechanical Leg

According to the analysis of the input and output of the mechanical leg in Section 2.2, the degrees of freedom of the mechanical leg in mammalian and insect modes are R*_X_*R*_Y_*T*_Z_* and R*_Z_*R*_Y_*T*_Z_*, respectively. According to the independent driving rule of the chain and the Jacobian matrix of the complete decoupled parallel mechanism, the output degrees of freedom of the moving platform controlled by the chain can be determined. In the mammalian model, the three chains control the moving platform’s rotation around the *X*-axis, rotation around the *Y*-axis, and translation along the *Z*-axis, respectively. In the insect mode, the three chains control the moving platform’s rotation around the *Y*-axis, rotation around the *Z*-axis, and translation along the *Z*-axis, respectively.

**Figure 5:**
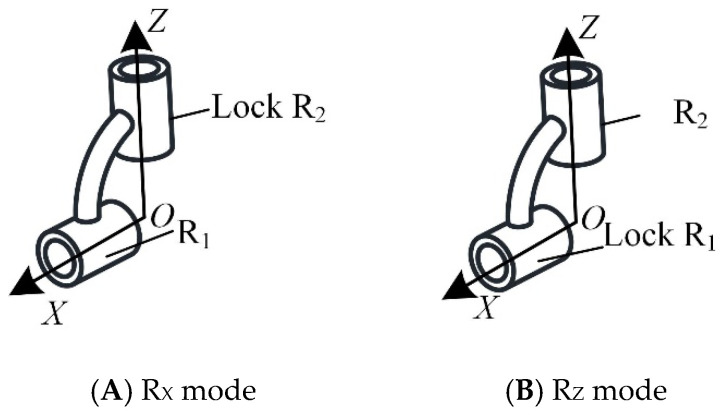
Lockable universal pair.

In this mechanism, the number of degrees of freedom and characteristics of chains I and II remain unchanged in the two motion modes. The chain with variable degrees of freedom is chain III. Chains I and II, respectively, control the translation of the moving platform along the *Z*-axis and the rotation of the moving platform around the *Y*-axis. Chain III controls the rotation of the moving platform around the *X*-axis and *Z*-axis in the two motion modes, respectively. According to the selection principle of the driving pair of the decoupled parallel mechanism, the axis of the driving pair of chain III is parallel to the *X*-axis and *Z*-axis, respectively, in the two motion modes. Therefore, the reconfigurable kinematic pair can be set as a lockable universal pair (Ur), which consists of two rotation axes, R_1_ and R_2_, as shown in Figure 5. When the mechanical leg is in the mammalian mode, R_2_ is locked, U*_r_* is in the R*_X_* mode, and R_1_ provides the rotational drive in the *X*-axis direction; when the mechanical leg is in the insect mode, R_1_ is locked, U*_r_* is in the R*_Z_* mode, and R_2_ provides the rotational drive in the *Z*-axis direction.

## 3. Chain Type Synthesis Process of Mechanism

### 3.1. Configuration Synthesis of Chain I

Chain I controls the moving platform’s translation along the *Z*-axis direction. The transmission wrench screw acting on the moving platform is a force line vector with one direction, always along the *Z*-axis direction.

When ***J***_dir_ and ***J***_inv_ satisfy the conditions of a diagonal matrix, ***J*** must be a diagonal matrix, and the mechanism is a completely decoupled mechanism. Therefore, [***J***_dir_]_11_ in the first row of ***J***_dir_ must be the only non-zero element in that row, so ***$***_T1_ can only be

(11) $${\$}_{T1}=(\begin{array}{lllllll} 0 & 0 & 1 & ; & 0 & 0 & 0 \end{array})$$

According to the selection principle of the driving pair of the decoupled parallel mechanism [24], chain I has three types of driving pairs: a prismatic pair along the *Z*-axis, a revolute pair with the axis parallel to the *X*-axis, and a revolute pair with the axis parallel to the *Y*-axis. The twist expression of the driving pair is

(12) $$\begin{array}{lllllllll} {\$}_{111} & = & (0 & 0 & 0 & ; & 0 & 0 & 1)\\ {\$}_{112} & = & (1 & 0 & 0 & ; & 0 & Q_{112} & R_{112})\\ {\$}_{113} & = & (0 & 1 & 0 & ; & P_{113} & 0 & R_{113}) \end{array}$$

In the first case, substituting ***$***_T1_ and ***$***_111_ into [***J***_inv_]_111_ gives

(13) $${\left[J_{\mathrm{inv}}\right]}_{111}={\$}_{T1}\circ {\$}_{111}=1\neq 0$$

At this time, [***J***_inv_]_111_ is always non-zero and meets the condition.

In the second case, substituting ***$***_T1_ and ***$***_112_ into [***J***_inv_]_112_ gives

(14) $${\left[J_{\mathrm{inv}}\right]}_{112}={\$}_{T1}\circ {\$}_{112}=R_{112}$$

In the third case, substituting ***$***_T1_ and ***$***_113_ into [***J***_inv_]_113_ gives

(15) $${\left[J_{\mathrm{inv}}\right]}_{113}={\$}_{T1}\circ {\$}_{113}=R_{113}$$

It can be seen from Equations (14) and (15) that *R*_112_ and *R*_113_ are parameters related to the position of the kinematic pair. As long as the position of the kinematic pair is adjusted so that *R*_112_ and *R*_113_ are not zero, [***J***_inv_]_11_ is non-zero in all three cases.

#### 3.1.1. The First Case

The twists ***$***_111_ and ***$***_T1_ of the chain I are

(16) $$\begin{array}{l} {\$}_{111}=(\begin{array}{lllllll} 0 & 0 & 0 & ; & 0 & 0 & 1 \end{array})\\ {\$}_{T1}=(\begin{array}{lllllll} 0 & 0 & 1 & ; & 0 & 0 & 0 \end{array}) \end{array}$$

The chains of the parallel mechanism must have the motion characteristics of the moving platform. The mechanical leg needs to switch between the R*_X_*R*_Y_*T*_Z_* and R*_Z_*R*_Y_*T*_Z_* motion modes. The chain’s degree-of-freedom type should include 1T3R, so the basic structure type of the first type of chain I is 1T3R. The underlined kinematic pair represents the driving pair. The subscripts *X*, *Y*, and *Z* represent the axial direction of the kinematic pair at the initial position. If two or more kinematic pairs have the same right-hand subscript letter, then the relative kinematic pairs are parallel to one another. Otherwise, they are orthogonal. P represents the prismatic pair, R represents the revolute pair, and the detailed chain structure is shown in the first category in Table 1. In order to simplify the structure and make the analysis intuitive, it is assumed that the axes of adjacent kinematic pairs are orthogonal or parallel.

#### 3.1.2. The Second Case

The twists ***$***_112_ and ***$***_T1_ of the chain I are

(17) $$\begin{array}{lllllllll} {\$}_{112} & = & (1 & 0 & 0 & ; & 0 & Q_{112} & R_{112})\\ {\$}_{T1} & = & (0 & 0 & 1 & ; & 0 & 0 & 0) \end{array}$$

When the driving pair is a revolute pair with the axis parallel to the *X*-axis direction, it is equivalent to replacing the translation drive along the *Z*-axis with a 2R parallel revolute pair. Therefore, the basic degree-of-freedom type for this form of chain I is 2T3R, and the specific chain structure is shown in the second category in Table 1.

#### 3.1.3. The Third Case

The twists ***$***_113_ and ***$***_T1_ of the chain I are

(18) $$\begin{array}{lllllllll} {\$}_{113} & = & (0 & 1 & 0 & ; & P_{113} & 0 & R_{113})\\ {\$}_{T1} & = & (0 & 0 & 1 & ; & 0 & 0 & 0) \end{array}$$

When the driving pair is a revolute pair with the axis parallel to the *Y*-axis direction, it is equivalent to replacing the translation drive along the *Z*-axis with a 2R parallel revolute pair. Therefore, the basic degree-of-freedom type for this form of chain I is 2T3R, and the specific chain structure is shown in the third case in Table 1.

Table 1 shows the possible structural types of chain I. It should be noted that in order to simplify the structure of the mechanism, the existence of redundant kinematic pairs is not considered for the chain described in this paper. In addition, due to space limitations, only one arrangement case is given for each type of chain. Changing the order of kinematic pairs in the chain is relatively simple, and such cases are not listed one by one.

### 3.2. Configuration Synthesis of Chain II

Chain II controls the moving platform’s rotation around the *Y*-axis. The transmission wrench screw ***$***_T2_ acting on the moving platform is a couple parallel to the *Y*-axis direction.

(19) $${\$}_{T2}=(\begin{array}{lllllll} 0 & 0 & 0 & ; & 0 & 1 & 0 \end{array})$$

The twist of the driving pair with a rotation component around the *X*-axis and *Z*-axis generates output that will affect the decoupled characteristics of the moving platform’s rotation around the *Y*-axis. The twist of the driving pair is

(20) $${\$}_{21}=(\begin{array}{lllllll} 0 & 1 & 0 & ; & P_{21} & 0 & R_{21} \end{array})$$

Thus,

(21) $${\left[J_{\mathrm{inv}}\right]}_{22}={\$}_{T2}\circ {\$}_{21}=1\neq 0$$

According to the characteristics of the chain’s degrees of freedom, chain II’s structure can be obtained, as shown in Table 2.

### 3.3. Configuration Synthesis of Chain III

Chain III can be equivalent to a lockable universal pair in series with other kinematic pairs, which is used to control the rotation of the moving platform around the *X*-axis and *Z*-axis in the mammalian and insect modes. For the convenience of analysis, the other kinematic pairs in the variable-degree-of-freedom chain, except for the lockable universal pair, are called constrained chains.

Chain III controls the rotation of the moving platform around the *X*-axis and *Z*-axis, respectively, in the two motion modes. When the mechanical leg is in the mammalian mode, the transmission wrench screw acting on the moving platform via the variable-degree-of-freedom chain ***$***_T31_ is a couple parallel to the *X*-axis. When the mechanical leg is in the insect mode, the transmission wrench screw acting on the moving platform via the chain with variable degrees of freedom ***$***_T32_ is a couple parallel to the *Z*-axis, which is expressed as

(22) $$\begin{array}{l} {\$}_{T31}=(\begin{array}{lllllll} 0 & 0 & 0 & ; & 1 & 0 & 0 \end{array})\\ {\$}_{T32}=(\begin{array}{lllllll} 0 & 0 & 0 & ; & 0 & 0 & 1 \end{array}) \end{array}$$

It can be seen that the twist of the lockable universal pair in the mammalian mode is ***$***_311_. The twist of the lockable universal pair in the insect mode is ***$***_312_, which is expressed as

(23) $$\begin{array}{l} {\$}_{311}=(\begin{array}{lllllll} 1 & 0 & 0 & ; & 0 & Q_{311} & R_{311} \end{array})\\ {\$}_{312}=(\begin{array}{lllllll} 0 & 0 & 1 & ; & P_{312} & Q_{312} & 0 \end{array}) \end{array}$$

When the mechanical leg is in the mammalian model, [***J***_inv_]_331_ of chain III is

(24) $${\left[J_{\mathrm{inv}}\right]}_{331}={\$}_{T31}\circ {\$}_{311}=1\neq 0$$

When the mechanical leg is in the insect mode, [***J***_inv_]_332_ of chain III is

(25) $${\left[J_{\mathrm{inv}}\right]}_{332}={\$}_{T32}\circ {\$}_{312}=1\neq 0$$

It can be seen from Equations (24) and (25) that [***J***_inv_]_331_ and [***J***_inv_]_332_ are always non-zero, which meets the requirements of the Jacobian matrix for the fully decoupled parallel mechanism.

Because the reciprocal product of the transmission wrench screw and the twist system of the constraint chain is 0 and the motion characteristics of the chain include the motion characteristics of the moving platform, in the configuration synthesis process of the constrained chains, attention should be paid to the following:

(1) The motion characteristics of the constraint chain do not include rotation around the *X*-axis and *Z*-axis, but they must include rotation around the *Y*-axis.
(2) The intersection between the motion characteristics of the constrained chain and those of chains I and II is the translation along the *Z*-axis.

It can be seen from the analysis that there are three cases—2, 3, and 4 degrees of freedom—for the constrained chain. According to the screw-theory-based method, the constrained chain structure can be obtained via configuration synthesis of the constrained chain, as shown in Table 3.

### 3.4. Feasible Constraint Model

The moving platform is subjected to a constraint force ${\$}_{F1}^{r}$ along the *X*-axis direction and a constraint force ${\$}_{F2}^{r}$ along the *Y*-axis direction in both the mammalian and insect modes. In the mammalian mode, the moving platform is subjected to a constraint couple ${\$}_{C1}^{r}$ around the *Z*-axis. In the insect mode, the moving platform is subjected to a constraint couple ${\$}_{C2}^{r}$ around the *X*-axis.

When a chain provides a constraint, the chain’s constraint type [25] may be

(26) $$A={\$}_{F_{1}}^{r};\;B={\$}_{F_{2}}^{r};\;C={\$}_{C_{1}}^{r}/{\$}_{C_{2}}^{r}$$

When a chain provides two constraints, the chain’s constraint type may be

(27) $$\left\{\begin{array}{l} D=AB={\$}_{F_{1}}^{r}\cup {\$}_{F_{2}}^{r}\\ E=AC={\$}_{F_{1}}^{r}\cup {\$}_{C_{1}}^{r}/{\$}_{C_{2}}^{r}\\ F=BC={\$}_{F_{2}}^{r}\cup {\$}_{C_{1}}^{r}/{\$}_{C_{2}}^{r} \end{array}\right.$$

where ∪ represents the union operation.

When a chain provides three constraints, the chain’s constraint type may be

(28) $$G=ABC={\$}_{F_{1}}^{r}\cup {\$}_{F_{2}}^{r}\cup {\$}_{C_{1}}^{r}/{\$}_{C_{2}}^{r}$$

When the chain is unconstrained, its constraint type is

(29) H=O

In the configuration synthesis of the mechanical leg, a constraint force and a constraint couple exist in each chain at the same time. The geometric conditions of the space required are complex, so a feasible constraint mode with only three independent constraints is selected. The feasible constraint modes that meet these requirements are *ABC*, *CDH*, *AEH*, and *GHH*. The constraint characteristics of each chain meeting requirements in different feasible constraint modes are shown in Table 4.

### 3.5. Selection of Feasible Constraint Mode

The mechanical leg is a 1T2R-type parallel mechanism in the mammalian and insect modes. It may generate parasitic motion in the rotation process, which increases the difficulty and complexity of planning the mechanism’s trajectory. Based on the topology design theory of the parallel mechanism, this study analyzes the motion characteristics of the moving platform in the four feasible constraint modes determined in Section 3.4.

The POC set calculation equations [26] for series and parallel mechanisms are

(30) $$M_{b}=\overset{m}{\underset{i=1}{\cup }}M_{J_{i}}$$

(31) $$M_{Pa}=\overset{v+1}{\underset{i=1}{\cap }}M_{b_{i}}$$

where $M_{J_{i}}$ is the POC set of the *i*-th kinematic pair; $M_{b}$ is the POC set of the serial chain; *M_Pa_* is the POC set of the moving platform of the mechanism; $M_{b_{i}}$ is the POC set of the end of the *i*-th chain; *m* is the number of kinematic pairs; and *v* is the number of independent loops.

Because the motion characteristics of the moving platform relative to the static platform are related to the position of the base point, the center point of the moving platform is generally selected as the reference point of a moving coordinate system; thus, the center point of the moving platform is selected as the base point. The center point *O*_1_ of the moving platform is positioned on the axis of the kinematic pair at the tail end of the chain with the fewest degrees of freedom among the three chains, which will reduce the occurrence of non-independent motion characteristics. Therefore, the base point *O*_1_ is located on the axis of the kinematic pair at the end of the chain with the fewest degrees of freedom.

#### 3.5.1. Motion Characteristics of Moving Platform in ABC Constraint Mode

The degree-of-freedom type for chains I and II is 2T3R, and the base point *O*_1_ can be located on the axis of the kinematic pair at the end of chain I or chain II.

The base point *O*_1_ is located on the axis of the end pair of chain I. The POC set of the chain I is

(32) $$M_{1}=\left[\begin{array}{c} t^{2}\left(⊥R_{11}\right)\\ r^{3} \end{array}\right]$$

where *t* and *r* represent translational and rotational degrees of freedom, respectively, and the superscript is the number of translational or rotational degrees of freedom.

The base point *O*_1_ is located on the axis of the end pair of chain II. The POC set of the chain II is

(33) $$M_{2}=\left[\begin{array}{c} t^{2}\cup \left\{t^{1}\left(⊥\rho \right)\right\}\\ r^{3} \end{array}\right]$$

where {*} denotes the parasitic translations induced by rotations.

The degree-of-freedom type for chain III is 3T2R, and its POC set is

(34) $$M_{3}=\left[\begin{array}{c} t^{3}\\ r^{2} \end{array}\right]$$

As can be seen from Equations (32)–(34), *M_Pa_* is

(35) $$M_{pa}=M_{1}\cap M_{2}\cap M_{3}=\left[\begin{array}{c} t^{2}\\ r^{3} \end{array}\right]\cap \left[\begin{array}{c} t^{2}\cup \left\{t^{1}\left(⊥\rho \right)\right\}\\ r^{3} \end{array}\right]\cap \left[\begin{array}{c} t^{3}\\ r^{2} \end{array}\right]=\left[\begin{array}{c} t^{2}\left(⊥R_{11}\right)\\ r^{2} \end{array}\right]$$

where *t*^2^(⊥R_11_) denotes that there are two finite translations in the plane perpendicular to the axis of R_11_.

Given that the mechanism has three DOFs, the POC set has only three independent elements, and any three of the four elements of *M_pa_* can be taken as independent elements. Thus, the mechanism has one dependent parasitic motion.

#### 3.5.2. Motion Characteristics of Moving Platform in CDH Constraint Mode

It can be seen from Table 4 that the degree-of-freedom type for chain I or chain II is 1T3R. When the degree-of-freedom type for chain I is 1T3R, the base point *O*_1_ is located on the axis of the end pair of chain I. The POC set of chain I is

(36) $$M_{1}=\left[\begin{array}{c} t^{1}\left(∥Z\right)\\ r^{3} \end{array}\right]$$

When the degree-of-freedom type for chain II is 1T3R, the base point *O*_1_ is located on the axis of the end pair of chain II. The POC set of chain II is

(37) $$M_{2}=\left[\begin{array}{c} t^{2}\left(⊥R_{21}\right)\\ r^{3} \end{array}\right]$$

where *t*^2^(⊥R_21_) denotes that there are two finite translations in the plane perpendicular to the axis of R_21_.

When the base point *O*_1_ is located on the axis of the end pair of chain I, the number of independent elements of the POC set for the end member is equal to the number of degrees of freedom of the chain.

When the degree-of-freedom type for chain I is 1T3R, that for chain II is 3T3R, and its POC set is

(38) $$M_{2}=\left[\begin{array}{c} t^{3}\\ r^{3} \end{array}\right]$$

The constraint type of chain III is C. Similarly, the POC set of chain III is

(39) $$M_{3}=\left[\begin{array}{c} t^{3}\\ r^{2} \end{array}\right]$$

As can be seen from Equations (36), (38) and (39), *M_Pa_* is

(40) $$M_{pa}=M_{1}\cap M_{2}\cap M_{3}=\left[\begin{array}{c} t^{1}\left(∥Z\right)\\ r^{3} \end{array}\right]\cap \left[\begin{array}{c} t^{3}\\ r^{3} \end{array}\right]\cap \left[\begin{array}{c} t^{3}\\ r^{2} \end{array}\right]=\left[\begin{array}{c} t^{1}\left(∥Z\right)\\ r^{2} \end{array}\right]$$

Given that the mechanism has three DOFs, the POC set has three independent elements, and the moving platform has no dependent parasitic motion.

#### 3.5.3. Motion Characteristics of Moving Platform in AEH Constraint Mode

The constraint type of chain III is E, the degree-of-freedom type is 2T2R, and the base point *O*_1_ is located on the axis of the end pair of chain III. The POC set of chain III is

(41) $$M_{3}=\left[\begin{array}{c} t^{2}\\ r^{2} \end{array}\right]$$

The constraint type of chain I is A, and the degree-of-freedom type is 2T3R. The POC set of chain I is

(42) $$M_{1}=\left[\begin{array}{c} t^{2}\cup \left\{t^{1}\left(⊥\rho \right)\right\}\\ r^{3} \end{array}\right]$$

The constraint type of chain II is H, and the degree-of-freedom type is 3T3R. The POC set of chain II is

(43) $$M_{2}=\left[\begin{array}{c} t^{3}\\ r^{3} \end{array}\right]$$

As can be seen from Equations (41)–(43), *M_Pa_* is

(44) $$M_{pa}=M_{1}\cap M_{2}\cap M_{3}=\left[\begin{array}{c} t^{2}\\ r^{2} \end{array}\right]\cap \left[\begin{array}{c} t^{2}\cup \left\{t^{1}\left(⊥\rho \right)\right\}\\ r^{3} \end{array}\right]\cap \left[\begin{array}{c} t^{3}\\ r^{3} \end{array}\right]=\left[\begin{array}{c} t^{2}\\ r^{2} \end{array}\right]$$

Given that the mechanism has three DOFs, the POC set has only three independent elements, and any three of the four elements of *M_pa_* can be taken as independent elements. The mechanism has one dependent parasitic motion.

#### 3.5.4. Motion Characteristics of Moving Platform in GHH Constraint Mode

The constraint type of chain III is G, the degree-of-freedom property is 1T2R, and the base point *O*_1_ is located on the axis of the end pair of chain III. The base point *O*_1_ is not located on the axis of the lockable universal pair [26], so the POC set of chain III is

(45) $$M_{3}=\left[\begin{array}{c} t^{1}\left(∥P_{31}\right)\cup \left\{t^{1}\left(⊥\rho \right)\right\}\\ r^{2} \end{array}\right]$$

The POC set of chain I is

(46) $$M_{1}=\left[\begin{array}{c} t^{3}\\ r^{3} \end{array}\right]$$

The POC set of chain II is

(47) $$M_{2}=\left[\begin{array}{c} t^{3}\\ r^{3} \end{array}\right]$$

As can be seen from Equations (45)–(47), *M_Pa_* is

(48) $$M_{pa}=M_{1}\cap M_{2}\cap M_{3}=\left[\begin{array}{c} t^{1}\left(∥P_{31}\right)\cup \left\{t^{1}\left(⊥\rho \right)\right\}\\ r^{2} \end{array}\right]\cap \left[\begin{array}{c} t^{3}\\ r^{3} \end{array}\right]\cap \left[\begin{array}{c} t^{3}\\ r^{3} \end{array}\right]=\left[\begin{array}{c} t^{1}\left(∥P_{31}\right)\cup \left\{t^{1}\left(⊥\rho \right)\right\}\\ r^{2} \end{array}\right]$$

Given that the mechanism has three DOFs, the POC set has only three independent elements, and any three of the four elements of *M_pa_* can be taken as independent elements. The mechanism has one dependent parasitic motion.

According to this analysis, in the CDH constraint mode, the moving platform has three independent elements in the POC set in the two motion modes. *M_pa_* indicates that there is no dependent parasitic motion on the moving platform. Therefore, CDH is chosen as the constraint mode of the mechanical leg.

### 3.6. Extension of Chain Structure Type

**Figure 6:**
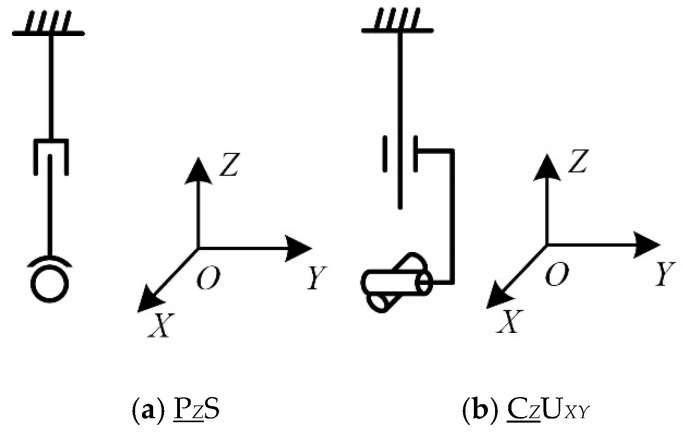
Structure diagram of chain I.

**Figure 7:**
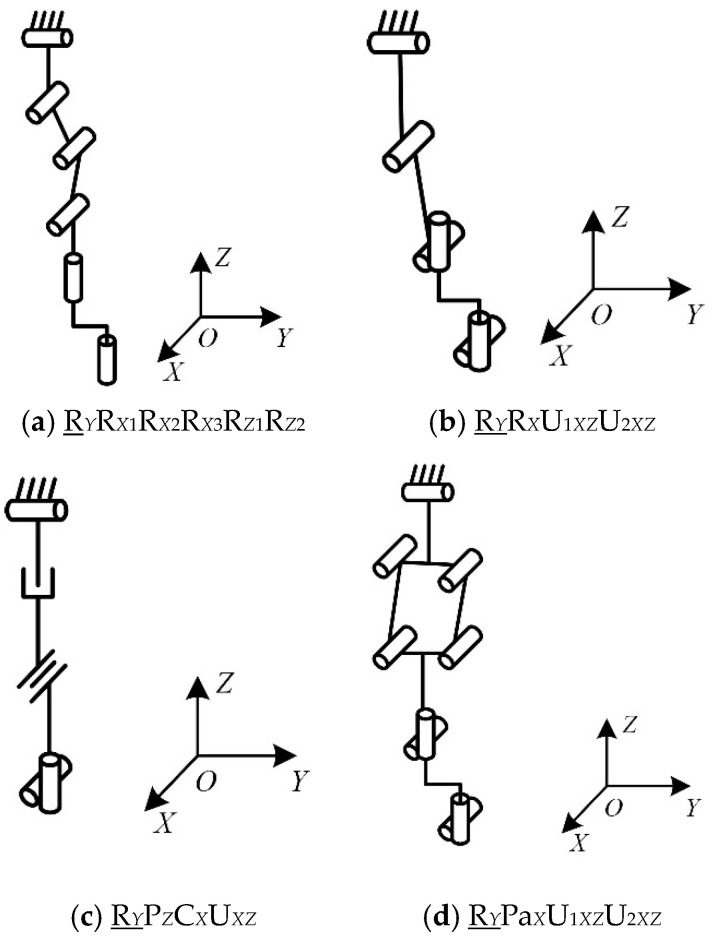
Structure diagram of chain II.

**Figure 8:**
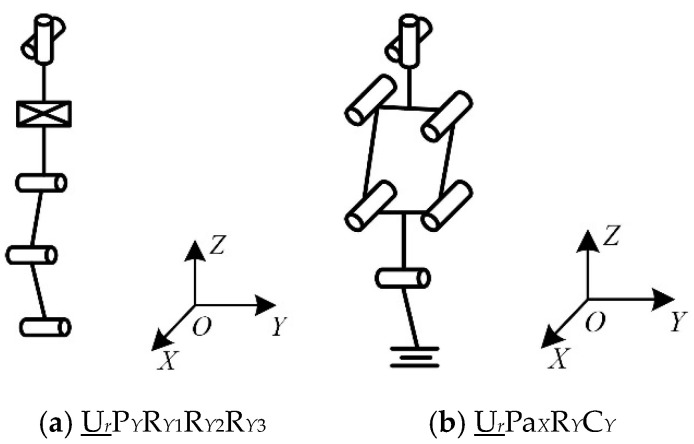
Structure diagram of chain III.

The chain can combine a single-degree-of-freedom kinematic pair into a multi-degree-of-freedom kinematic pair. For example, the revolute pair and the prismatic pair can be combined into a cylindric pair, the spherical pair can replace a plurality of revolute pairs, and the single-degree-of-freedom kinematic pair can be combined into a composite kinematic pair, such as a Pa pair. The structure of the extended chain is shown in Table 5, Table 6 and Table 7. The configurations of the parts of chains I, II, and III are shown in Figure 6, Figure 7 and Figure 8.

## 4. Evaluation of Mechanical Leg Mechanism Configuration

In order to ensure the performance and use effect of the mechanical leg, it is necessary to evaluate and optimize the configuration of each chain of the mechanical leg [27,28]. In the design process of a parallel mechanism, the aim is to ensure that it has a compact structure and can be easily protected while also reducing the difficulty of prototype design. The factors that affect the compactness of the structure, the difficulty of protection, and the difficulty of prototype fabrication mainly depend on the selection of kinematic pairs.

The fabrication difficulty *SC*_1_ of the chain in the mechanism can be expressed as

(49) $$SC_{1}=\frac{k_{p}n_{P}+k_{R}n_{R}+k_{U}n_{U}+k_{S}n_{S}+k_{\mathrm{Pa}}n_{\mathrm{Pa}}+k_{C}n_{C}}{n}$$

where *n*_P_, *n*_R_, *n*_U_, *n*_S_, *n*_Pa_, and *n*_C_ represent the number of prismatic pairs, revolute pairs, universal pairs, spherical pairs, Pa pairs, and cylindrical pairs in the chain; *N* is the total number of kinematic pairs, *n* = *n*_P_ + *n*_R_ + *n*_U_ + *n*_S_ + *n*_Pa_ + *n*_C_; and *k*_P_, *k*_R_, *k*_U_, *k*_S_, *k*_Pa_, and *k*_C_ are difficulty indices of kinematic pair fabrication.

The smaller the value of the fabrication difficulty index *SC*_1_, the easier the fabrication of the kinematic pair. The fabrication of the revolute and prismatic pairs is relatively easy, so *k*_P_ and *k*_R_ are taken as 1. The spherical and universal pairs can be produced through the continuous combination of revolute pairs, so *k*_U_ and *k*_S_ are taken as 2. The Pa and cylindrical pairs have strict requirements for installation position and rod length in the manufacturing process, so *k*_Pa_ and *k*_C_ are taken as 3.

The compactness *SC*_2_ of the chain in the mechanism can be expressed as

(50) $$SC_{2}=\frac{j_{p}n_{P}+j_{R}n_{R}+j_{U}n_{U}+j_{S}n_{S}+j_{\mathrm{Pa}}n_{\mathrm{Pa}}+j_{C}n_{C}}{n}$$

where *j*_P_, *j*_R_, *j*_U_, *j*_S_, *j*_Pa_, and *j*_C_ are the compactness indices of kinematic pairs.

A small value of the compactness index *SC*_2_ indicates that the chain is more compact. Spherical and universal pairs have more compact structures and more degrees of freedom, so the value of *j*_U_ and *j*_S_ is 1. If all the required degrees of freedom of the chain are realized by the revolute pair, the overall volume of the mechanism will be huge, so *j*_R_ and *j*_C_ are taken as 2. The Pa pair is a planar closed-loop kinematic pair composed of four coaxial revolute pairs, which can replace a basic prismatic pair. The mechanical structure of the moving pair is generally large and complex, so *j*_Pa_ and *j*_P_ are taken as 3.

During movement, the mechanical leg is often impacted and rubbed by other objects, so it is necessary to select kinematic pairs that are easy to protect in their design [27]. The degree of difficulty of protecting the chain of the mechanism, *SC*_3_, is

(51) $$SC_{3}=\frac{f_{p}n_{P}+f_{R}n_{R}+f_{U}n_{U}+f_{S}n_{S}+f_{\mathrm{Pa}}n_{\mathrm{Pa}}+f_{C}n_{C}}{n}$$

where *f*_P_, *f*_R_, *f*_U_, *f*_S_, *f*_Pa_, and *f*_C_ are indices of the degree of difficulty of protecting the kinematic pair.

The smaller the value of the protection difficulty index *SC*_3_, the easier the chain’s protection. The structure of the revolute pair is compact and easy to protect [29]. The spherical and universal pairs have more degrees of freedom and compact structures, so *f*_R_, *f*_U_, and *f*_S_ are taken as 1. The prismatic pair usually consists of a sliding block and a guide rail, and the latter is usually intermittently exposed to the outside during the operation of the prismatic pair. The guard rail surface is very easily damaged when hit or scratched by other objects, and it is not easy to protect. The Pa pair can be equivalent to a prismatic pair. Therefore, *f*_Pa_ and *f*_P_ are taken as 2. The cylindrical pair can be equivalent to a prismatic pair and a revolute pair, and the axes of the two pairs coincide, so *f*_C_ is taken as 3.

As can be seen from Equations (49)–(51), the smaller the values of the three indices *SC*_1_, *SC*_2_, and *SC*_3_, the lower the complexity of the representative mechanism in the manufacturing and protection processes. Considering the influence of the three factors, the evaluation index *WSC*_DM_ of the complexity of the structure is defined as

(52) $$WSC_{DM}=w_{1}SC_{1}+w_{2}SC_{2}+w_{3}SC_{3}$$

where *w*_1_, *w*_2_, and *w*_3_ are the weights of *SC*_1_, *SC*_2_, and *SC*_3_, respectively, with *w*_1_ = 0.3, *w*_2_ = 0.4, and *w*_3_ = 0.3.

From the analysis in Section 3, the complexity of the chains in different combinations can be obtained, as shown in Table 8, Table 9, Table 10 and Table 11. 

When chain I is P_Z_S, its complexity is 1.7, which is the smallest value for chain I that meets the requirements. When chain II is R*_Y_*U_1*XZ*_U_2*XZ*_R*_Z_* or R*_Y_*U_1*XZ*_U_2*XZ*_R*_X_*, its complexity is 1.35, which is the smallest value for chain II that meets the requirements. When chain Ⅲ is U*_r_*R*_Y_*_1_R*_Y_*_2_R*_Y_*_3_P*_Y_*, its complexity is 1.54, which is the smallest value for chain Ⅲ that meets the requirements.

**Figure 9:**
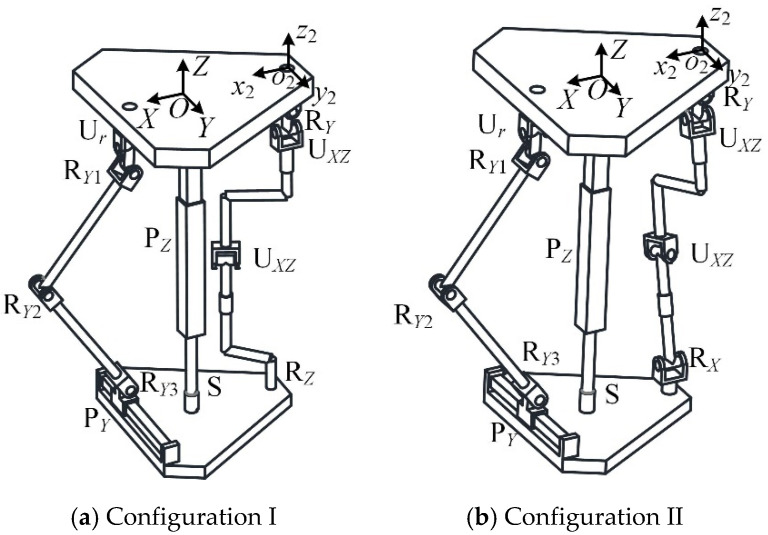
Reconfigurable bionic mechanical leg.

The reconfigurable mechanical leg has two configurations: in configuration I, chain II is R*_Y_*U_1*XZ*_U_2*XZ*_R*_Z_*, as shown in Figure 9a, and in configuration II, chain II is R*_Y_*U_1*XZ*_U_2*XZ*_R*_X_*, as shown in Figure 9b.

When the reconfigurable mechanical leg is in configuration I, the chain coordinate system *o*_2_-*x*_2_*y*_2_*z*_2_ is established, where the *y*_2_ axis is parallel to the R*_Y_* axis of chain II and the *z*_2_ axis is oriented upward. If the R*_Z_* axis coincides with the U_1*XZ*_ axis, chain II will have a passive degree of freedom. The twist of the chain II is

(53) $$\begin{array}{lllllllll} {\$}_{211} & = & (0 & 1 & 0 & ; & d_{1} & 0 & 0)\\ {\$}_{221} & = & (1 & 0 & 0 & ; & 0 & e_{2} & f_{2})\\ {\$}_{231} & = & (0 & 0 & 1 & ; & d_{3} & e_{3} & 0)\\ {\$}_{241} & = & (1 & 0 & 0 & ; & 0 & e_{4} & f_{4})\\ {\$}_{251} & = & (0 & 0 & 1 & ; & d_{5} & e_{5} & 0)\\ {\$}_{261} & = & (0 & 0 & 1 & ; & d_{3} & e_{3} & 0) \end{array}$$

Here, ***$***_231_ and ***$***_261_ are linearly dependent, and there is a reciprocal screw. Chain II is no longer an unconstrained chain and does not meet the requirements of the feasible constraint mode. Therefore, configuration II is selected as the configuration of the reconfigurable mechanical leg.

**Figure 10:**
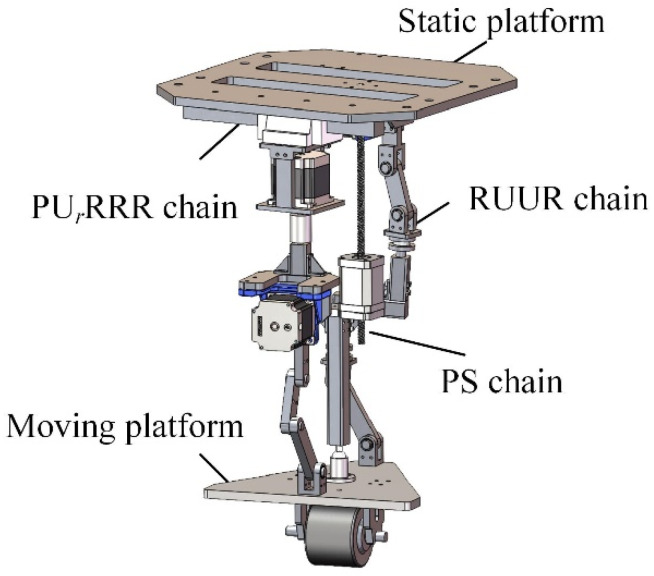
Three-dimensional model of mechanical leg.

**Figure 11:**
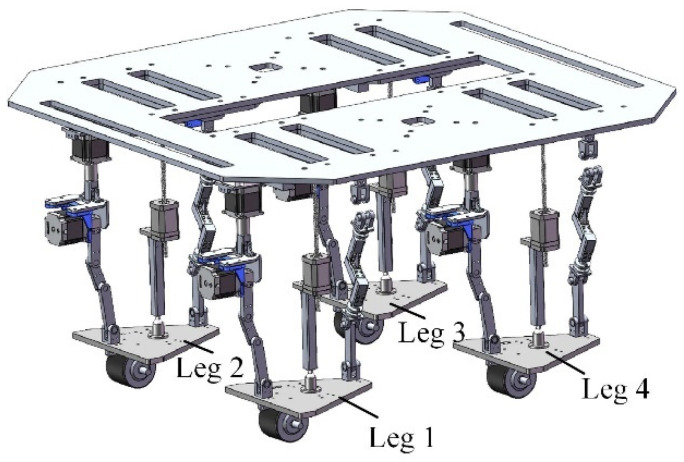
Three-dimensional model of robot.

The sequence and layout of the kinematic pairs have an important influence on the performance and manufacturing complexity of the mechanical leg. From the analysis in Section 3, it can be seen that the only arrangement order of chain I is P*_Z_*S. There is no need to adjust the kinematic pair sequence of chain I. The arrangement order of chain II is R*_Y_*R*_X_*U_1*XZ*_U_2*XZ*_, R*_Y_*U_1*XZ*_R*_X_*U_2*XZ*_, or R*_Y_*U_1*XZ*_U_2*XZ*_R*_X_*. When chain II is R*_Y_*R*_X_*U_1*XZ*_U_2*XZ*_ or R*_Y_*U_1*XZ*_R*_X_*U_2*XZ*_, it is difficult to ensure that the rotation axis of R*_X_* is perpendicular to that of R*_Y_* during movement. Therefore, the arrangement order of chain II is R*_Y_*U_1*XZ*_U_2*XZ*_R*_X_*. The order of chain III is U*_r_*P*_Y_*R*_Y_*_1_R*_Y_*_2_R*_Y_*_3_, U*_r_*R*_Y_*_1_R*_Y_*_2_R*_Y_*_3_P*_Y_*, or P*_Y_*U*_r_*R*_Y_*_1_R*_Y_*_2_R*_Y_*_3_. When chain III is U*_r_*P*_Y_*R*_Y_*_1_R*_Y_*_2_R*_Y_*_3_ or U*_r_*R*_Y_*_1_R*_Y_*_2_R*_Y_*_3_P*_Y_*, the motion range of the kinematic pair is limited by the chain or the moving platform. When installed on the moving platform, the horizontal prismatic pair will increase the weight and inertia of the moving platform and increase the difficulty of its motion control. When chain III is P*_Y_*U*_r_*R*_Y_*_1_R*_Y_*_2_R*_Y_*_3_, the horizontal prismatic pair is installed on the static platform, so the high stiffness and stability of the static platform can be used to reduce the motion error caused by the deformation of the chain or the moving platform. A wide range of horizontal translation can be achieved, thereby expanding the mechanism’s working space. Therefore, P*_Y_*U*_r_*R*_Y_*_1_R*_Y_*_2_R*_Y_*_3_ is selected as the arrangement order of chain III. The parallel mechanical leg is a PS + RUUR + PU*_r_*RRR reconfigurable parallel mechanism, and its model diagram is shown in Figure 10. The three-dimensional model of the reconfigurable four-wheel-legged robot is shown in Figure 11, where the legs are numbered.

## 5. Kinematic Analysis of the Reconfigurable Mechanical Leg

### 5.1. Inverse Position Solution

Obtaining the inverse position solution entails solving each driving parameter by determining the position vector of the end point of the foot in the fixed coordinate system. The closed-loop vector method and the Denavit–Hartenberg (D-H) parameter method are used to establish the inverse position equations of the mechanical leg in the mammalian and insect modes.

**Figure 12:**
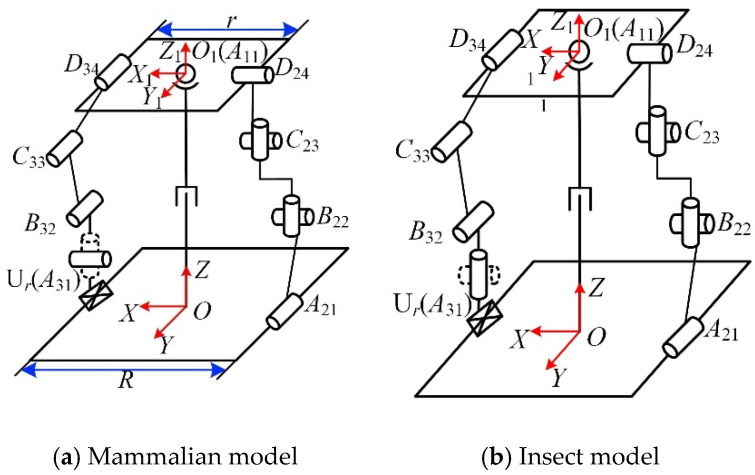
Schematic diagram of reconfigurable parallel mechanism.

Firstly, the inverse position solution of chain I is analyzed by using the closed-loop vector method. The position vector of the center point *O*_1_ of the moving platform in the static coordinate system *O*-*XYZ* is ***OO***_1_ = [0 0 *z*]^T^, and the center point *A*_11_ of the spherical pair is set to coincide with *O*_1_, as shown in Figure 12. The position vector of point *A*_11_ in the moving coordinate system *O*_1_-*X*_1_*Y*_1_*Z*_1_ is [0 0 0]^T^.

The attitude transformation matrix ***T*** of the moving coordinate system relative to the static coordinate system is

(54) $$\begin{array}{l} T=\mathrm{Rot}\left(Z,\gamma \right)\mathrm{Rot}\left(Y,\beta \right)\mathrm{Rot}\left(X,\alpha \right)\\ \;\;\;=\left[\begin{array}{ccc} c\alpha c\beta & c\alpha s\beta s\gamma -s\alpha c\gamma & c\alpha s\beta c\gamma +s\alpha s\gamma \\ s\alpha c\beta & s\alpha s\beta s\gamma +c\alpha c\gamma & s\alpha s\beta c\gamma -c\alpha s\gamma \\ -s\beta & c\beta s\gamma & c\beta c\gamma \end{array}\right] \end{array}$$

where s represents sin, c represents cos, and *γ*, *β*, and *α* are the rotation angles of the moving platform around the *Z*-axis, *Y*-axis, and *X*-axis, respectively.

The position vector of $A_{11}^{O_{1}}$ is transformed into the static coordinate system *O*-*XYZ* through the attitude transformation matrix ***T*** as follows:

(55) $$OA_{11}^{O}=TO_{1}A_{11}^{O_{1}}+OO_{1}={\left[\begin{array}{ccc} 0 & 0 & z \end{array}\right]}^{T}$$

The position vector ***l***_11_ of chain I can be expressed as

(56) $$l_{11}=OA_{11}^{O}={\left[\begin{array}{ccc} 0 & 0 & z \end{array}\right]}^{T}$$

**Figure 13:**
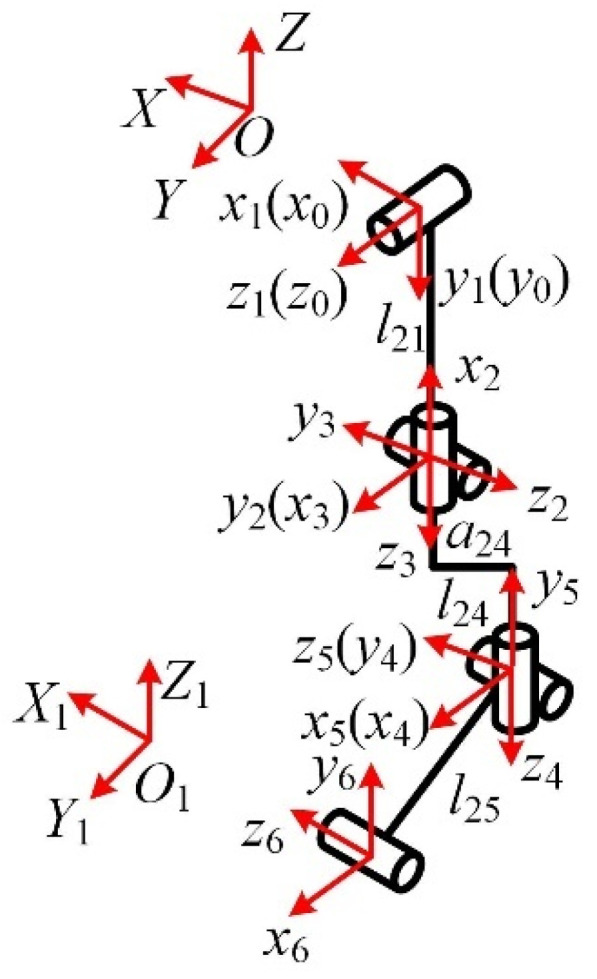
DH coordinate system of chain II.

The D-H parameter method is used to obtain the inverse position solution of chain II. The D-H coordinate system of chain II is established, as shown in Figure 13, and the corresponding D-H parameters are shown in Table 12.

*α_i_*_-1_ is the degree of rotation from *z_i_*_-1_ to *z_i_* about the *x_i_*_-1_ axis; *a_i_*_-1_ is the length of movement from *z_i_*_-1_ *to z_i_* in the direction of the *x_i_*_-1_ axis; *θ_i_* is the number of degrees of rotation from *x_i_*_-1_ to *x_i_* about the *z_i_ axis*; and *di* is the length of movement from *x_i_*_-1_ to *x_i_* in the direction of the *z_i_* axis.

The data in Table 12 are substituted into the following matrix transformation equation:

(57) Tii−1=cθi-sθi0ai−1sθicαi−1cθicαi−1-sαi−1−disαi−1sθisαi−1cθisαi−1cαi−1dicαi−10001

**Figure 14:**
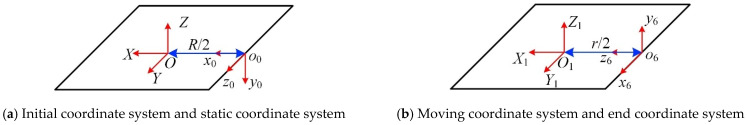
Chain II coordinate system and static and moving coordinate systems.

The positional relationship between the initial coordinate system *o*_0_-*x*_0_*y*_0_*z*_0_ of chain II and the static coordinate system *O*-*XYZ* is shown in Figure 14a. The positional relationship between the moving coordinate system *O*_1_-*X*_1_*Y*_1_*Z*_1_ and chain II’s end coordinate system *o*_6_-*x*_6_*y*_6_*z*_6_ is shown in Figure 14b.

The transformation matrix T20O from the initial coordinate system *o*_0_-*x*_0_*y*_0_*z*_0_ of chain II to the static coordinate system *O*-*XYZ* is

(58) T20O=100−R/200100−1000001

The transformation matrix T2O16 from the moving coordinate system *O*_1_-*X*_1_*Y*_1_*Z*_1_ to chain II’s end coordinate system *o*_6_-*x*_6_*y*_6_*z*_6_ is

(59) T2O16=01000010100r/20001

The transformation matrix T2O1O from the moving platform to the static platform can be obtained as follows:

(60) T2O1O=T20OT210T221T232T243T254T265T2O16=M112M122M132M142M212M222M232M242M312M322M332M342M412M422M432M442

In the mammalian mode, the transformation matrix $T_{YX}^{2}$ of the moving platform is

(61) $$T_{YX}^{2}=\left[\begin{array}{cccc} c\beta & s\alpha s\beta & c\alpha s\beta & x\\ 0 & c\alpha & -s\alpha & y\\ -s\beta & c\beta s\alpha & c\alpha c\beta & z\\ 0 & 0 & 0 & 1 \end{array}\right]$$

In the insect mode, the transformation matrix $T_{YZ}^{2}$ of the moving platform is

(62) $$T_{YZ}^{2}=\left[\begin{array}{cccc} c\beta c\gamma & -c\beta s\gamma & s\beta & x\\ s\gamma & c\gamma & 0 & y\\ -c\gamma s\beta & s\beta s\gamma & c\beta & z\\ 0 & 0 & 0 & 1 \end{array}\right]$$

The transformation matrices T2O1O of chain II in the mammalian and insect modes are equal to the elements of the corresponding position of the transformation matrix of the moving platform.

In the mammalian mode,

(63) T2O1O=TYX2

In the insect mode,

(64) T2O1O=TYZ2

After simplified calculations, the solution of chain II’s driving *θ*_21_ can be obtained as follows:

(65) $$\theta_{21}=\beta$$

**Figure 15:**
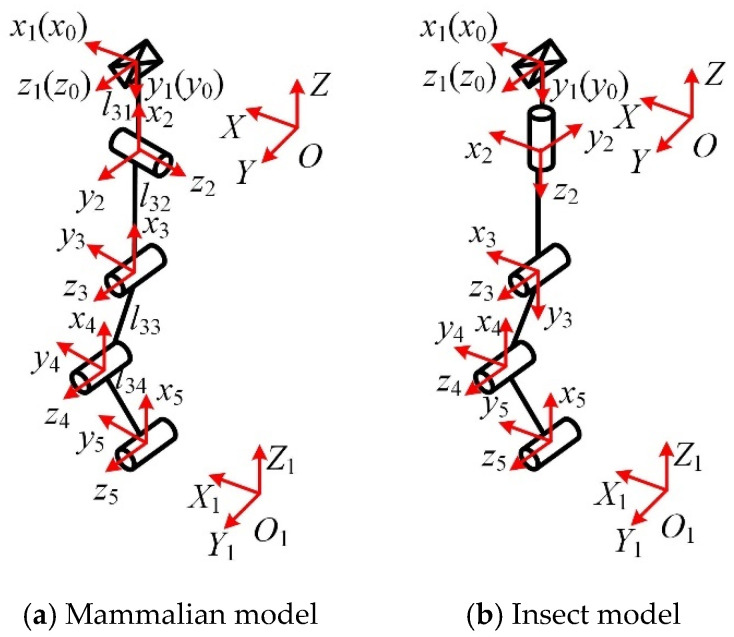
DH coordinate system of chain III.

The D-H parameter method is used to obtain the inverse position solution of chain III. The D-H coordinate system of chain III in the mammalian and insect modes is established, as shown in Figure 15.

The D-H parameters of chain III in the mammalian and insect models are shown in Table 13 and Table 14, respectively.

**Figure 16:**
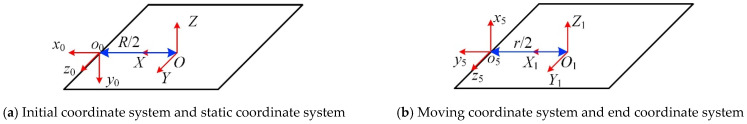
Chain III coordinate system and static and moving coordinate systems.

The positional relationship between the initial coordinate system *o*_0_-*x*_0_*y*_0_*z*_0_ of chain III and the static coordinate system *O*-*XYZ* is shown in Figure 16a; the positional relationship between the moving coordinate system *O*_1_-*X*_1_*Y*_1_*Z*_1_ and chain III’s end coordinate system *o*_5_-*x*_5_*y*_5_*z*_5_ is shown in Figure 16b.

The transformation matrix from the initial coordinate system *o*_0_-*x*_0_*y*_0_*z*_0_ of chain III to the static coordinate system *O*-*XYZ* is

(66) T30O=100R/200100−1000001

The transformation matrix from the moving coordinate system *O*_1_-*X*_1_*Y*_1_*Z*_1_ to chain III’s end coordinate system *o*_5_-*x*_5_*y*_5_*z*_5_ is

(67) T3O15=0010100r/201000001

The transformation matrix from the moving platform to the static platform can be obtained as follows:

(68) T3O1O=T30OT310T321T332T343T354T3O15=M113M123M133M143M213M223M233M243M313M323M333M343M413M423M433M443

In the mammalian mode, the transformation matrix $T_{XY}^{3}$ of the moving platform is

(69) $$T_{XY}^{3}=\left[\begin{array}{cccc} c\beta & 0 & s\beta & x\\ s\alpha s\beta & c\alpha & -s\alpha c\beta & y\\ -c\alpha s\beta & s\alpha & c\alpha c\beta & z\\ 0 & 0 & 0 & 1 \end{array}\right]$$

In the insect mode, the transformation matrix $T_{ZY}^{3}$ of the moving platform is

(70) $$T_{ZY}^{3}=\left[\begin{array}{cccc} c\beta c\gamma & -s\gamma & s\beta c\gamma & x\\ c\beta s\gamma & c\gamma & s\beta s\gamma & y\\ -s\beta & 0 & c\beta & z\\ 0 & 0 & 0 & 1 \end{array}\right]$$

The transformation matrices T3O1O of chain III in the mammalian and insect modes are equal to the elements of the corresponding position of the transformation matrix of the moving platform.

In the mammalian mode,

(71) T3O1O=TXY3

In the insect mode,

(72) T3O1O=TXY3

After simplified calculations, the driving solution of chain III in the mammalian mode can be obtained as follows:

(73) $$\theta_{32}=\alpha$$

In the mammalian mode, *d*_31_ is

(74) $$d_{31}=\left(Z-\frac{r}{2}c\alpha s\beta -l_{31}\right)t\alpha$$

where t represents tan.

After simplified calculations, the driving solution of chain III in insect mode can be obtained as follows:

(75) $$\theta_{32}=\gamma$$

In the insect mode, *d*_31_ is

(76) $$d_{31}=\frac{R}{2}t\gamma$$

### 5.2. Velocity Analysis

In this section, the Jacobian matrix of the mechanism in the two modes is obtained by deriving the inverse solution of the position of the mechanism in the mammalian and insect modes.

When the mechanical leg is in the mammalian mode, the derivatives of Equations (56), (65), and (73) with respect to time *t* are obtained:

(77) $$\left[\begin{array}{c} {\dot{l}}_{11}\\ {\dot{\theta }}_{32}\\ {\dot{\theta }}_{21} \end{array}\right]=J^{1}\left[\begin{array}{c} \dot{z}\\ \dot{\alpha }\\ \dot{\beta } \end{array}\right]=\left[\begin{array}{ccc} 1 & 0 & 0\\ 0 & 1 & 0\\ 0 & 0 & 1 \end{array}\right]\left[\begin{array}{c} \dot{z}\\ \dot{\alpha }\\ \dot{\beta } \end{array}\right]$$

where ***J***^1^ is the Jacobian matrix of the mechanism in the mammalian mode.

When the mechanical leg is in the insect mode, the derivatives of Equations (56), (65), and (75) with respect to time *t* are obtained:

(78) $$\left[\begin{array}{c} {\dot{l}}_{11}\\ {\dot{\theta }}_{21}\\ {\dot{\theta }}_{32} \end{array}\right]=J^{2}\left[\begin{array}{c} \dot{z}\\ \dot{\beta }\\ \dot{\gamma } \end{array}\right]=\left[\begin{array}{ccc} 1 & 0 & 0\\ 0 & 1 & 0\\ 0 & 0 & 1 \end{array}\right]\left[\begin{array}{c} \dot{z}\\ \dot{\beta }\\ \dot{\gamma } \end{array}\right]$$

where ***J***^2^ is the Jacobian matrix of the mechanism in the insect mode.

It can be seen from Equations (77) and (78) that the Jacobian matrix of the mechanical leg in both the mammalian and insect modes is a unit diagonal matrix. It is shown that the degrees of freedom of the reconfigurable mechanical leg are completely decoupled in the two motion modes.

## 6. Performance Analysis and Optimization Design of the Reconfigurable Mechanical Leg

### 6.1. Workspace Analysis

**Figure 17:**
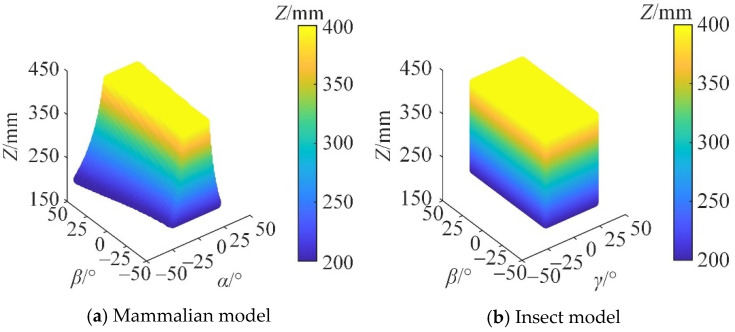
Three-dimensional diagram of workspace.

The working space of the mechanical leg determines the step length and the obstacle-crossing ability of the mobile robot. Its size and shape represent the range of motion of the mechanical leg’s end point, which is an important index for measuring the kinematical performance of the mechanical leg. The translation distance of the prismatic pair is [200, 500] mm, and the rotation range of the revolute pair is [−50, 50]°. The maximum rotation angle of the spherical pair is 50°. In the mammalian mode, the search space of the mechanical leg is Z ∈ [150, 500] mm, *α* ∈ [−60, 60]°, *β* ∈ [−60, 60]°; in the insect mode, the search space of the mechanical leg is Z ∈ [150, 500] mm, *β* ∈ [−60, 60]°, *γ* ∈ [−60, 60]°. Under the condition that the constraint conditions are satisfied, the workspace of the mechanical leg in the mammalian and insect modes is obtained, as shown in Figure 17.

It can be seen from Figure 17 that the reconfigurable mechanical leg has a large reachable working space range in both the mammalian and insect modes. The workspace is continuous and has no holes. When the reconfigurable mechanical leg is in the mammalian mode, the ranges of *α*, *β*, and *Z* are [−43, 43]°, [−45, 45]°, and [200, 400] mm, respectively. The rotation angle of the moving platform decreases with the increase in Z. When the reconfigurable mechanical leg is in the insect mode, the ranges of *γ*, *β*, and Z are [−23, 23]°, [−45, 45]°, and [200, 400] mm, respectively, which indicates a larger workspace than that in the mammalian mode. The shape is a regular cuboid, which is more suitable for fast walking over complex terrain.

The performance index *W*_V_ of the reachable workspace can be defined as

(79) $$W_{V}=\frac{W_{i}}{Q}$$

where *Q* is the number of points in the search space, and *W_i_* is the number of points satisfying the constraint condition.

The value range of WV is [0, 1], and the larger the value is, the larger the workspace volume is.

### 6.2. Motion/Force Transmission Performance Analysis

The motion/force transmission performance index of the structure comprises input and output transmission performance indices. The input transmission performance index is the result of the interaction between the input twist and the transmission wrench screw, which indicates the efficiency with which the driver’s input energy is transferred to the chain. The output transmission performance index is the result of the interaction between the transmission wrench screw and the output twist, which indicates the efficiency with which the force spiral on the chain is transferred to the moving platform.

The equations for calculating the input/output transfer performance indices of a non-redundant parallel mechanism [30,31] are

(80) $$\lambda_{i}=\frac{\left|{\$}_{Ti}\circ {\$}_{Ii}\right|}{{\left|{\$}_{Ti}\circ {\$}_{Ii}\right|}_{\max}}$$

(81) $$\eta_{i}=\frac{\left|{\$}_{Ti}\circ {\$}_{Oi}\right|}{{\left|{\$}_{Ti}\circ {\$}_{Oi}\right|}_{\max}}$$

(82) $$\Gamma =\min\left\{\lambda_{i},\eta_{i}\right\}$$

where *λ_i_* is the input transfer performance index of chain *i*, *η_i_* is the output transfer performance index of chain *i*, Γ is the local transfer performance index, ***$***_I*i*_ is the input twist of chain *i*, ***$***_T*i*_ is the transmission wrench screw of chain *i*, and ***$***_O*i*_ is the output twist of chain *i*.

The input and output transfer performance indices represent the transfer of energy from the drive input to the chain and from the chain to the output, respectively. According to the definition, the value ranges of *λ_i_*, *η_i_*, and Γ are between 0 and 1, and due to the dimensionless nature of the index, its size is independent of the selection of the coordinate system. The closer the value of Γ is to 1, the better the motion/force transmission performance of the mechanism is. On the contrary, it indicates that the mechanism is closer to singularity.

Taking the reconfigurable mechanical leg in the general configuration as the research object and taking chain I as an example, ***$***_I1_, ***$***_T1_, and ***$***_O1_ are calculated. The twist system of chain I in the general configuration is

(83) $$\begin{array}{lllll} {\$}_{11} & = & (0 & ; & Z)\\ {\$}_{12} & = & (X & ; & r_{A_{11}}\times X)\\ {\$}_{13} & = & (Y & ; & r_{A_{11}}\times Y)\\ {\$}_{14} & = & (Z & ; & r_{A_{11}}\times Z) \end{array}$$

The input twist ***$***_I1_ of chain I is the twist of the driving pair, specifically

(84) $${\$}_{I1}={\$}_{11}=\left(\begin{array}{ccc} 0 & ; & Z \end{array}\right)$$

where ***Z*** is the direction vector of the *Z*-axis.

Similarly, the input twists ***$***_I2_ and ***$***_I3_ of chains II and III are, respectively,

(85) $$\begin{array}{lllllll} {\$}_{I2} & = & {\$}_{21} & = & (Y & ; & r_{A_{21}}\times Y)\\ {\$}_{I3} & = & {\$}_{32} & = & (u_{32} & ; & r_{A_{31}}\times u_{32}) \end{array}$$

where ***Y*** is the direction vector of the *Y*-axis, $r_{A_{21}}$ and $r_{A_{31}}$ are the position vectors of *A*_21_ and *A*_31_, and ***u***_32_ is the unit vector of the rotation axis of the lockable universal pair.

Assuming that the driving pair P*_Z_* of chain I is locked, ***$***_11_ will be removed from the twist of chain I, and by taking the reciprocal product of the kinematic twist of the remaining kinematic pairs, the transmission wrench screw ***$***_T1_ of chain I is obtained as follows:

(86) $${\$}_{T1}=\left(\begin{array}{ccc} Z & ; & 0 \end{array}\right)$$

In the same way, the transmission wrench screw ***$***_T2_ of chain II can be obtained as follows:

(87) $${\$}_{T2}=\left(\begin{array}{ccc} 0 & ; & Y \end{array}\right)$$

When the reconfigurable mechanical leg is in the mammalian mode, the transmission wrench screw ***$***_T31_ of chain III is

(88) $${\$}_{T31}=\left(\begin{array}{ccc} 0 & ; & X \end{array}\right)$$

where ***X*** is the direction vector of the *X*-axis.

When the reconfigurable mechanical leg is in the insect mode, the transmission wrench screw ***$***_T32_ of chain III is

(89) $${\$}_{T32}=\left(\begin{array}{ccc} 0 & ; & Z \end{array}\right)$$

When the reconfigurable mechanical leg is in the mammalian mode, the drive of chains II and III is locked, whereas the drive of chain I is retained. Each chain can generate a five-dimensional constraining force wrench composed of $\left[\begin{array}{lllll} {\$}_{F_{1}}^{r}, & {\$}_{F_{2}}^{r}, & {\$}_{C_{1}}^{r}, & {\$}_{T2}, & {\$}_{T31} \end{array}\right]$ to act on the moving platform. In this case, the mechanism has a single degree of freedom, and the output twist ***$***_O11_ of the chain I is

(90) $${\$}_{O11}=\left(\begin{array}{ccccccc} 0 & 0 & 0 & ; & 0 & 0 & 1 \end{array}\right)$$

Similarly, the output twists ***$***_O21_ and ***$***_O31_ of chains II and III can be obtained as follows:

(91) $$\begin{array}{lllllllll} {\$}_{O21} & = & (0 & c\alpha & s\alpha & ; & -Z_{C1}c\alpha & 0 & 0)\\ {\$}_{O31} & = & (1 & 0 & 0 & ; & 0 & Z_{C1} & 0) \end{array}$$

where *Z_A_*_11_ is the coordinate of *A*_11_ on the *Z*-axis.

When the reconfigurable mechanical leg is in the insect mode, the output twist of each chain is

(92) $$\begin{array}{lllllllll} {\$}_{O12} & = & (0 & 0 & 0 & ; & 0 & 0 & 1)\\ {\$}_{O22} & = & (c\gamma & s\gamma & 0 & ; & -Z_{C1}s\gamma & Z_{C1}c\gamma & 0)\\ {\$}_{O32} & = & (0 & 0 & 1 & ; & 0 & 0 & 0) \end{array}$$

When the reconfigurable mechanical leg is in the mammalian mode, the input and output transmission indices of each chain are, respectively,

(93) $$\begin{array}{l} \lambda_{11}=\lambda_{21}=\lambda_{31}=1\\ \eta_{11}=\eta_{31}=1\\ \eta_{21}=\left|c\alpha \right| \end{array}$$

When the reconfigurable mechanical leg is in the insect mode, the input transmission index and the output transmission index of each chain are, respectively,

(94) $$\begin{array}{l} \lambda_{12}=\lambda_{22}=\lambda_{32}=1\\ \eta_{12}=\eta_{32}=1\\ \eta_{22}=\left|s\gamma \right| \end{array}$$

The local transmission index of the mechanical leg in the mammalian mode is

(95) $$\Gamma_{1}=\min\left\{\lambda_{11},\lambda_{21},\lambda_{31},\eta_{11},\eta_{21},\eta_{31}\right\}=\left|c\alpha \right|$$

The local transmission index of the mechanical leg in the insect mode is

(96) $$\Gamma_{2}=\min\left\{\lambda_{12},\lambda_{22},\lambda_{32},\eta_{12},\eta_{22},\eta_{32}\right\}=\left|s\gamma \right|$$

**Figure 18:**
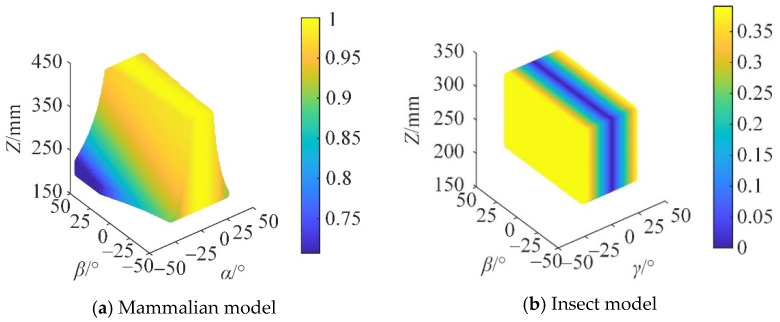
Distribution of Γ in the reachable workspace.

From Equations (95) and (96), the motion/force transmission performance index of the reconfigurable mechanical leg in the mammal and insect modes can be obtained, as shown in Figure 18.

In Figure 18, different colors represent the magnitude of the *Γ* value. When the reconfigurable mechanical leg is in the mammalian mode, Γ increases with the increase in *Z*. When the reconfigurable mechanical leg is in the insect mode, the fluctuation of Γ on the *β* axis is small, and the motion performance is stable.

**Figure 19:**
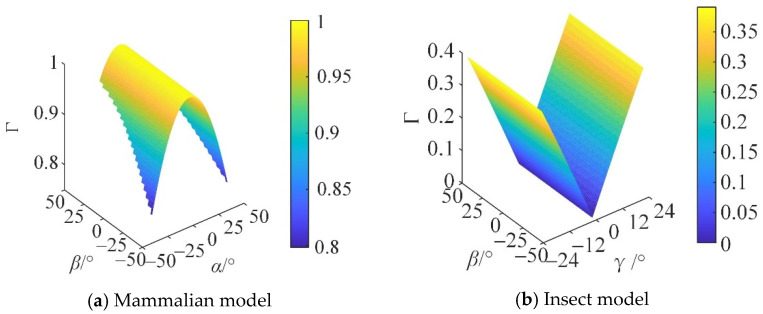
Γ distribution at Z = 250 mm.

The distribution of Γ in the plane cannot be directly observed in Figure 18. Therefore, the three-dimensional distribution of Γ in the section at *Z* = 250 mm is taken to present the variation in Γ within the workspace, as shown in Figure 19.

When the robot is subjected to external loads, the smaller the internal force required inside the robot, the better its motion/force performance, that is, the higher its carrying capacity. It can be seen from Figure 19 that at Z = 250 mm, the motion/force transmission performance of the mammalian model is significantly better than that of the insect model. In the high-load application scenario, the mammalian mode is selected to meet the task requirements.

As can be seen from Equation (82), Γ only reflects the motion/force transmission performance of the parallel mechanism at a certain point in the workspace. It cannot be used to evaluate the global motion/force transmission performance of the mechanism. Therefore, in the workspace of the parallel mechanism, the global motion/force transmission performance evaluation index $\bar{\zeta }$ is defined as

(97) $$\bar{\zeta }=\frac{\sum_{i=1}^{W_{i}}\Gamma }{W_{i}}$$

From the definition of $\tilde{\zeta }$, the global motion/force transfer performance index is an average of the local motion/force transfer performance index Γ over the entire workspace, such that its size reflects the average transmission performance of the mechanism in the whole working space. However, it cannot reflect the fluctuation of the mechanism’s performance across the whole working space. During the movement of the reconfigurable mechanical leg, the mechanism is usually expected to have good average performance while maintaining stability. Based on the weighted standard deviation of motion/force transmission performance, a global motion performance fluctuation index of the parallel mechanism is proposed in this paper. The equation for evaluating the degree of fluctuation of the mechanism’s performance index $\tilde{\zeta }$ is

(98) $$\tilde{\zeta }=\frac{\sum_{i=1}^{W_{i}}\Gamma \sqrt{{\left(\Gamma -\bar{\zeta }\right)}^{2}}}{W_{i}}$$

According to the definition of standard deviation, the larger the standard deviation $\tilde{\zeta }$, the larger the fluctuation of the mechanism’s transmission performance in the working space. A smaller $\tilde{\zeta }$ indicates more stable motion/force performance of the mechanism in the workspace.

From Equations (95) and (96), it can be seen that when the reconfigurable mechanical leg is in the mammalian mode, the performance index of global motion/force transmission $\bar{\zeta }$ is 0.9752, and $\tilde{\zeta }$ is 0.0212. When the reconfigurable mechanical leg is in the insect mode, the global motion/force transmission performance index $\bar{\zeta }$ is 0.2021, and $\tilde{\zeta }$ is 0.0201, indicating that when subjected to external loads, the mammalian configuration requires less transmission force than the insect configuration.

Through a comparative analysis of the workspace and motion/force transmission performance of the reconfigurable mechanical leg in the mammalian and insect modes, it can be seen that the insect mode is superior to the mammalian mode in terms of the workspace; in terms of motion/force transmission performance, the mammalian model is stronger than the insect model. Therefore, for the reconfigurable four-wheel-legged robot, if a large and stable carrying capacity is required during the movement, the mammalian mode is selected, whereas the insect mode is selected if a large step size is required during movement.

### 6.3. Optimized Design

In this section, the workspace and motion/force transfer performance of the reconfigurable mechanical leg in the two motion modes are taken as the objective function, and the particle swarm optimization algorithm is used to optimize the geometric size parameters of the mechanism.

#### 6.3.1. Design Variables

Based on the above analysis, the performance index of the reconfigurable mechanical leg is related to the side length *R* of the static platform and the length of the links *l*_31_ and the side length *r* of the moving platform. Therefore, the parameters for dimension optimization are *R*, *l*_31_, and *r*.

#### 6.3.2. Objective Function

In the process of optimization design, the superiority of the mechanism’s performance and stability in the workspace should be taken into account. Therefore, in the kinematical optimization design of the mechanism, $\bar{\zeta }$ and $\tilde{\zeta }$ should be considered comprehensively, with the aim of making $\bar{\zeta }$ as large as possible and $\tilde{\zeta }$ as small as possible. Therefore, the comprehensive kinematic performance index *ζ* of the mechanism is proposed, that is,

(99) $$\zeta =\left(1-\tilde{\zeta }\right)\bar{\zeta }$$

For the parallel mechanism, under the premise of meeting design requirements, it is often desirable to maximize the reachable workspace for optimal performance. From Equations (79) and (99), it can be seen that the workspace performance index and the comprehensive kinematic performance index range from 0 to 1. The larger the value of the two, the larger the workspace, and the better the transmission performance of the mechanical leg. When the reconfigurable mechanical leg is optimized, the reference value of the objective function of the workspace and the comprehensive kinematic performance index is set to 1 so that the objective function is minimized. The single objective optimization function of the workspace performance index and the comprehensive kinematic performance index of the reconfigurable mechanical leg in the mammalian and insect modes is

(100) $$\left\{\begin{array}{l} f_{1}=1-W_{V1}\\ f_{2}=1-\zeta_{1}\\ f_{3}=1-W_{V2}\\ f_{4}=1-\zeta_{2} \end{array}\right.$$

where *W_V_*_1_ and *ζ*_1_ are the performance indices in the mammalian mode, and *W_V_*_2_ and *ζ*_2_ are those in the insect mode.

The reference target distance method can be used to transform the four objective functions into a single objective function. This method is similar to the multi-objective approach. The same direction processing is simple and widely used. Using this method, the objective function can be defined as

(101) $$F\left(x\right)=\frac{1}{4}\left(\sum_{i=1}^{4}\left|Z_{i}-f_{i}\left(X\right)\right|\right)$$

where *Z_i_* is the reference value of the objective function and is set as 1.

#### 6.3.3. Constraints

According to the structural parameters of the mechanical leg, the constraint range of each design parameter is given, as shown in Table 15.

#### 6.3.4. Optimization Examples

The parameters of the particle swarm optimization algorithm are shown in Table 16. The optimization results for the mechanism’s size parameters and performance indices are shown in Table 17 and Table 18, respectively.

It can be seen from Table 17 that *R* and *l*_31_ are increased, while *r* is reduced. Because the precision and manufacturing cost of the mechanism will be limited in the actual processing, the design parameters of the optimized mechanism in Table 17 are rounded, and the results are *R* = 440 mm, *l*_31_ = 140 mm, and *r* = 120 mm. The rounded size parameters are substituted into the workspace and comprehensive motion performance index formulas for the two modes. The optimized mechanism performance indices can be obtained, as shown in Table 18. In the mammalian mode, the workspace index of the mechanism is increased by 76.4%, and the comprehensive motion performance index is reduced by 5.0%. In the insect mode, the workspace index of the mechanism is increased by 4.3%, and the comprehensive motion performance index is increased by 3.9%.

#### 6.3.5. Comparison of Results

**Figure 20:**
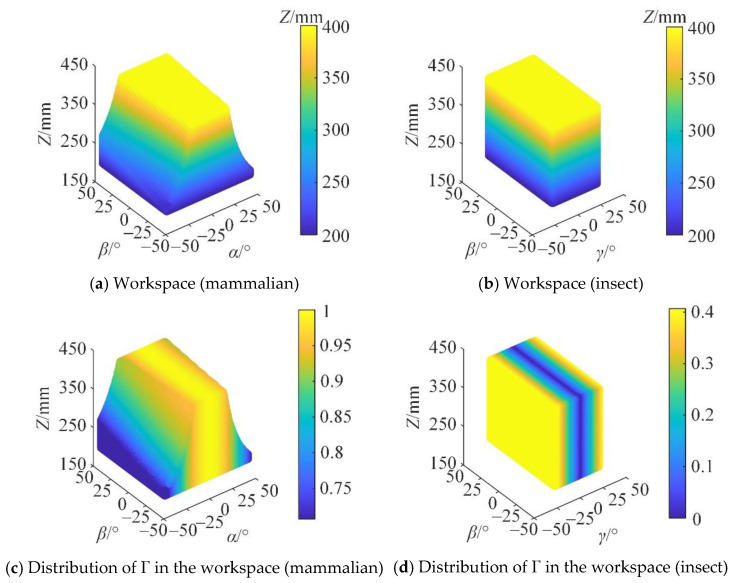
Performance indices after mechanism optimization.

According to the optimized structural parameters, the kinematic performance index of the mechanism is recalculated, as shown in Figure 20.

Figure 20 shows that, compared with the size before optimization, the workspace range has increased and the comprehensive motion performance index has decreased. In the insect mode, the workspace performance and motion/force transmission performance are improved compared with before optimization. The overall kinematic performance index of the mechanical leg is improved.

## 7. Research on Stability of Reconfigurable Four-Wheel-Legged Robot

**Figure 21:**
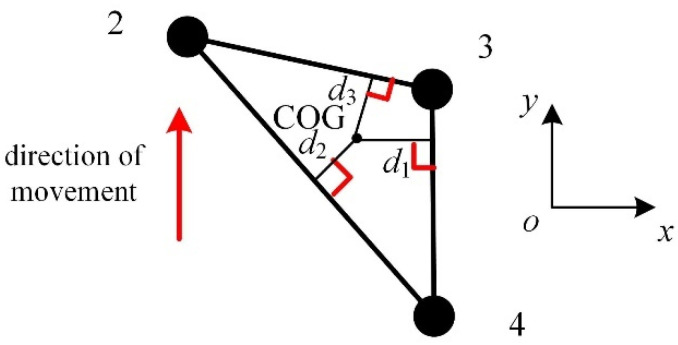
Static stability margin of the robot.

In the walking operation mode, the reconfigurable four-wheel-legged robot needs to have a large step space so that it can adapt to a complex and changeable unstructured terrain. As shown in Section 6, the volume of the workspace of the mechanical leg in the insect mode is larger than that in the mammalian mode. Therefore, when the robot is in the walking mode, it is better to use the insect mode as the configuration of the reconfigurable mechanical leg. It is necessary to analyze the robot’s stability while moving so that it remains stable during movement. When the quadruped robot enters the static walking mode, one leg is used as the swing leg, and the other three legs are used as support legs. The robot’s center of mass must always be located in the triangular support area composed of the three support legs, as shown in Figure 21.

The stability margin *d_M_* [32] of the robot is

(102) $$d_{M}=\min\left\{\begin{array}{ccc} d_{1}, & d_{2}, & d_{3} \end{array}\right\}$$

where *d_i_* is the distance from the center of gravity to these three sides.

When *d_M_* > 0, the robot is in a stable state; when *d_M_* = 0, the robot is in a critical state, and a slight external disturbance can cause the body to become imbalanced; and when *d_M_* < 0, the robot is in an unstable state.

**Figure 22:**
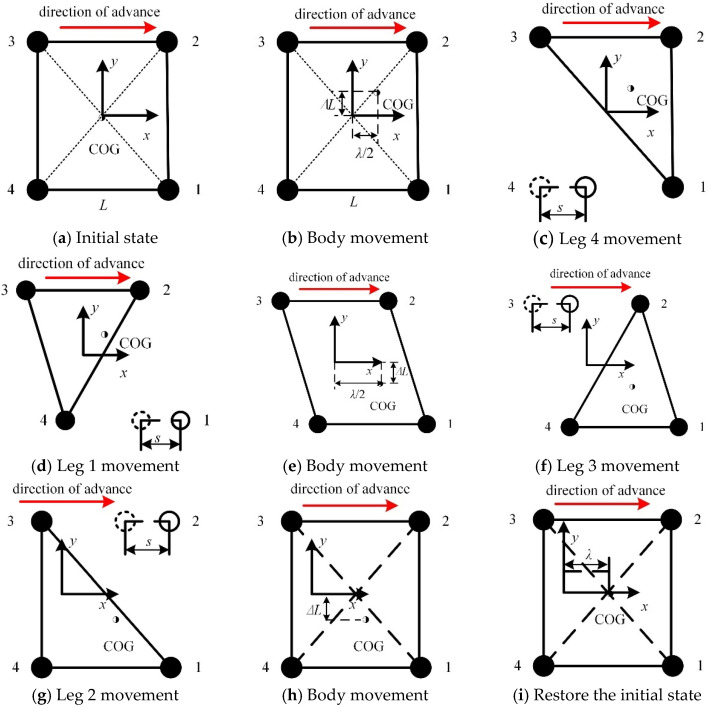
Center-of-mass adjustment in the 4-1-3-2 gait of the robot.

In the static gait, there are A44=24 kinds of robot steps, among which the 4-1-3-2 gait is the best step order for a quadruped robot in static gait [33]. As the robot moves, it is necessary to adjust the position of its center of gravity to prevent it from overturning. In Figure 22, ◌ represents the foot drop point at the previous moment, ● represents the foot drop point, ○ represents the point where the foot is about to fall, ◑ represents the center of gravity of the body, *L* is the distance between the center points of adjacent foot ends, *s* is the robot step size, and *λ* is the stride. The static gait of the robot is planned, as shown in Figure 22.

Before the robot walks, the four legs are in an upright state, as shown in Figure 22a. The center of gravity of the fuselage is located at the geometric center of the quadrilateral support area composed of legs 1, 2, 3, and 4.

In order to ensure that the robot remains static and stable when leg 4 swings, the body’s center of gravity needs to be adjusted before leg 4 moves. The body moves *λ*/2 forward along the *x*-axis and moves ∆*L* upward along the *y*-axis, as shown in Figure 22b.

The robot moves leg 4 and moves *s* along the *x*-axis. The body’s center of gravity is located in the triangular support area composed of legs 1, 2, and 3, as shown in Figure 22c.

The robot moves leg 1 and moves *s* along the *x*-axis. The body’s center of gravity is located in the triangular support area composed of legs 2, 3, and 4, as shown in Figure 22d.

In order to ensure that the robot remains static and stable when leg 3 swings, the body’s center of gravity needs to be adjusted before leg 3 moves. The body continues to move *λ*/2 forward along the *x*-axis and moves 2∆*L* downward along the *y*-axis, as shown in Figure 22e.

The robot moves leg 3 and moves *s* along the *x*-axis. The body’s center of gravity is located in the triangular support area composed of legs 1, 2, and 4, as shown in Figure 22f.

The robot moves leg 2 and moves *s* along the *x*-axis. The body’s center of gravity is located in the triangular support area composed of legs 1, 3, and 4, as shown in Figure 22g.

After leg 2 touches the ground, the body moves up ∆*L* along the *y*-axis, and the robot returns to the initial position, as shown in Figure 22h.

The body’s center of gravity moves forward along the *x*-axis with a stride *λ*. The center of gravity is located at the geometric center of the quadrilateral support area composed of legs 1, 2, 3, and 4 as support legs, as shown in Figure 22i.

## 8. Conclusions

(1) Based on bionic principles and configuration synthesis theory for a decoupled parallel mechanism, a method for configuration synthesis of a reconfigurable decoupled mechanical leg was proposed. The mechanical leg switches between mammalian and insect modes through a change in the lockable universal pair rotating shaft.
(2) Based on the difficulty of fabricating and protecting the chain, as well as its compactness, an evaluation index for the complexity of the chain of the reconfigurable mechanical leg was proposed, and a series of synthesized chain configurations were evaluated. Then, the configuration of each chain of the reconfigurable mechanical leg was determined.
(3) Based on the weighted standard deviation of motion/force transmission performance, a global performance fluctuation index of the motion/force transmission of the mechanical leg was proposed. The proposed index can reflect the fluctuation of the mechanism’s motion performance in the workspace and evaluate its stability.
(4) When the robot is in the walking operation mode, the insect mode is used as the configuration of the reconfigurable mechanical leg. The static stability criterion is used to plan the robot’s gait such that it can meet the needs of the task environment.

## Figures and Tables

**Table 1:** Structure of Basic Chain I.

Driving Category	Characteristics of Degrees of Freedom	Kinematic Pair Type	Chain Type
The first case	1T3R	1P3R	P_Z_R_X_R_Y_R_Z_
	2T3R	2P3R	P_Z_R_X_R_Y_R_Z_P_X_; P_Z_R_X_R_Y_R_Z_P_Y_
		1P4R	P_Z_R_X_R_Y_R_Z1_R_Z2_
	3T3R	3P3R	P_Z_P_X_P_Y_R_X_R_Y_R_Z_
		2P4R	P_Z_P_X_R_X_R_Y_R_Z1_R_Z2_; P_Z_P_Y_R_X_R_Y_R_Z1_R_Z2_
		1P5R	P_Z_R_X_R_Y_R_Z1_R_Z2_R_Z3_;
The second case	2T3R	4R1P	R_X1_R_X2_R_Y_R_Z_P_Y_
		5R	R_X1_R_X2_R_X3_R_Y_R_Z_
	3T3R	4R2P	R_X1_R_X2_R_Y_R_Z_P_X_P_Y_
		5R1P	R_X1_R_X2_R_X3_R_Y_R_Z_P_X_; R_X1_R_X2_R_Y1_R_Y2_R_Z_P_Y_ R_X1_R_X2_R_Y_R_Z1_R_Z2_P_Y_; R_X1_R_X2_R_Y_R_Z1_R_Z2_P_X_
		6R	R_X1_R_X2_R_Y_R_Z1_R_Z2_R_Z3_; R_X1_R_X2_R_X3_R_Y_R_Z1_R_Z2_ R_X1_R_X2_R_Y1_R_Y2_R_Z1_R_Z2_; R_X1_R_X2_R_X3_R_Y1_R_Y2_R_Z_
The third case	2T3R	4R1P	R_Y1_R_Y2_R_X_R_Z_P_X_
		5R	R_Y1_R_Y2_R_X_R_Z1_R_Z2_
	3T3R	4R2P	R_Y1_R_Y2_R_X_R_Z_P_X_P_Y_
		5R1P	R_Y1_R_Y2_R_X_R_Z1_R_Z2_P_Y_; R_Y1_R_Y2_R_X_R_Z1_R_Z2_P_X_
		6R	R_Y1_R_Y2_R_X_R_Z1_R_Z2_R_Z3_

**Table 2:** Structure of Basic Chain II.

Connectivity	Characteristics of Degrees of Freedom	Kinematic Pair Type	Chain Type
4	1T3R	3R1P	R_Y_P_Z_R_X_R_Z_
5	2T3R	3R2P	R_Y_P_Y_P_Z_R_X_R_Z_; R_Y_P_X_P_Z_R_X_R_Z_
		4R1P	R_Y_P_Y_R_X1_R_X2_R_Z_; R_Y_P_Z_R_X1_R_X2_R_Z_
		5R	R_Y_R_X1_R_X2_R_X3_R_Z_
6	3T3R	3R3P	R_Y_P_X_P_Y_P_Z_R_X_R_Z_
		4R2P	R_Y_P_Y_P_X_R_X1_R_X2_R_Z_; R_Y_P_Z_P_X_R_X1_R_X2_R_Z_; R_Y_P_Y_P_Z_R_X_R_Z1_R_Z2_; R_Y_P_X_P_Z_R_X_R_Z1_R_Z2_
		5R1P	R_Y_P_Y_R_X1_R_X2_R_Z1_R_Z2_; R_Y_P_X_R_X1_R_X2_R_X3_R_Z_; R_Y_P_X_R_X1_R_X2_R_Z1_R_Z2_; R_Y_P_Z_R_X1_R_X2_R_Z1_R_Z2_; R_Y_P_Z_R_X_R_Z1_R_Z2_R_Z3_
		6R	R_Y1_R_X1_R_X2_R_Z1_R_Z2_R_Z3_; R_Y_R_X1_R_X2_R_X3_R_Z1_R_Z2_

**Table 3:** Constrained chain structure.

Connectivity	Characteristics of Degrees of Freedom	Kinematic Pair Type	Chain Type
2	1T1R	1P1R	P_Z_R_Y_
3	2T1R	2P1R	P_X_P_Z_R_Y_; P_Z_P_X_R_Y_; P_Z_P_Y_R_Y_
		1P2R	P_X_R_Y1_R_Y2_; P_Z_R_Y1_R_Y2_; R_Y1_P_Y_R_Y2_
		3R	R_Y1_R_Y2_R_Y3_
4	3T1R	3P1R	P_X_P_Y_P_Z_R_Y_
		2P2R	P_Y_P_X_R_Y1_R_Y2_; P_Y_P_Z_R_Y1_R_Y2_
		3R1P	P_Y_R_Y1_R_Y2_R_Y3_

**Table 4:** Constraint characteristics of each chain in feasible constraint mode.

Feasible Constraint Pattern	Chain	Degree-of-Freedom Type	Constraint Type
ABC	1	2T3R	A/B
	2	2T3R	B/A
	3	3T2R	C
CDH	1	1T3R/3T3R	D/H
	2	3T3R/1T3R	H/D
	3	3T2R	C
AEH	1	2T3R/3T3R	A/H
	2	3T3R/2T3R	H/A
	3	2T2R	E
GHH	1	3T3R	H
	2	3T3R	H
	3	1T2R	G

**Table 5:** Structure of chain I.

Chain	Basic Chain Structure	Chain Structure with Multi-Degree-of-Freedom Kinematic Pair	Chain Structure Containing Closed-Loop Structures
Chain I	P_Z_R_X_R_Y_R_Z_	P_Z_S; C_Z_U_XY_	_—_

**Table 6:** Structure of chain II.

Chain	Basic Chain Structure	Chain Structure with Multi-Degree-of-Freedom Kinematic Pair	Chain Structure Containing Closed-Loop Structures
Chain II	R_Y_P_X_P_Y_P_Z_R_X_R_Z_	R_Y_P_X_P_Y_P_Z_U_XZ_	R_Y_P_X_P_Y_Pa_Y_U_XZ_; R_Y_P_X/Y_Pa_X/Y_Pa_X/Y_U_XZ_; R_Y_Pa_Y/X_Pa_X/Y_Pa_X/Y_U_XZ_
	R_Y_P_X_P_Y_P_Z_R_X_R_Z_	C_Y_P_X_P_Z_U_XZ_; C_Y_C_Z_C_X_	C_Y_P_X/Z_Pa_Y_U_XZ_; C_Y_Pa_Y_Pa_Y_U_XZ_
	R_Y_P_Y_P_X_R_X1_R_X2_R_Z_	R_Y_P_X_P_Y_R_X_U_XZ_; R_Y_P_Y_C_X_U_XZ_; C_Y_C_X_U_XZ_	R_Y_P_X_Pa_Z_R_X_U_XZ_; R_Y_Pa_Z_Pa_Z_R_X_U_XZ_; R_Y_Pa_Z_C_X_U_XZ_
	R_Y_P_Z_P_X_R_X1_R_X2_R_Z_	R_Y_P_Z_P_X_R_X_U_XZ_; R_Y_P_Z_C_X_U_XZ_; R_Y_R_X_C_Z_C_X_	R_Y_P_Z_Pa_Y_R_X_U_XZ_; R_Y_Pa_Y_Pa_Y_R_X_U_XZ_; R_Y_Pa_Y_C_X_U_XZ_
	R_Y_P_Y_P_Z_R_X_R_Z1_R_Z2_	R_Y_P_Y_P_Z_R_Z_U_XZ_; R_Y_P_Y_C_Z_U_XZ_; C_Y_C_Z_U_XZ_	R_Y_P_Y_Pa_X_R_Z_U_XZ_; R_Y_Pa_X_Pa_X_R_Z_U_XZ_; R_Y_Pa_X_C_Z_U_XZ_
	R_Y_P_X_P_Z_R_X_R_Z1_R_Z2_	R_Y_P_X_P_Z_R_Z_U_XZ_; R_Y_P_X_C_Z_U_XZ_; R_Y_R_Z_C_X_C_Z_	R_Y_P_X_Pa_Y_R_Z_U_XZ_; R_Y_Pa_Y_Pa_Y_R_Z_U_XZ_; R_Y_Pa_Y_C_Z_U_XZ_
	R_Y_P_Y_R_X1_R_X2_R_Z1_R_Z2_	R_Y_P_Y_R_X_R_Z_U_XZ_; R_Y_P_Y_U_1XZ_U_2XZ_; C_Y_P_Y_U_1XZ_U_2XZ_	R_Y_Pa_X/Z_R_X_R_Z_U_XZ_; R_Y_Pa_X/Z_U_1XZ_U_2XZ_;
	R_Y_P_X_R_X1_R_X2_R_X3_R_Z_	R_Y_P_X_R_X1_R_X2_U_XZ_; R_Y_R_X_C_X_U_XZ_;	R_Y_Pa_Y/Z_R_X1_R_X2_U_XZ_
	R_Y_P_X_R_X1_R_X2_R_Z1_R_Z2_	R_Y_P_X_R_X_R_Z_U_XZ_; R_Y_P_X_U_1XZ_U_2XZ_; R_Y_R_Z_C_X_U_XZ_	R_Y_Pa_Y/Z_R_X_R_Z_U_XZ_; R_Y_Pa_Y/Z_U_1XZ_U_2XZ_;
	R_Y_P_Z_R_X1_R_X2_R_Z1_R_Z2_	R_Y_P_Z_R_X_R_Z_U_XZ_; R_Y_P_Z_U_1XZ_U_2XZ_; R_Y_R_X_C_Z_U_XZ_	R_Y_Pa_Y/X_R_X_R_Z_U_XZ_; R_Y_Pa_Y/X_U_1XZ_U_2XZ_
	R_Y_P_Z_R_X_R_Z1_R_Z2_R_Z3_	R_Y_P_Z_R_Z1_R_Z2_U_XZ_; R_Y_C_Z_R_Z_U_XZ_	R_Y_Pa_Y/X_R_Z1_R_Z2_U_XZ_
	R_Y_R_X1_R_X2_R_Z1_R_Z2_R_Z3_	R_Y_R_X_R_Z1_R_Z2_U_XZ_; R_Y_R_Z_U_1XZ_U_2XZ_	_—_
	R_Y_R_X1_R_X2_R_X3_R_Z1_R_Z2_	R_Y_R_Z_R_X1_R_X2_U_XZ_; R_Y_R_X_U_1XZ_U_2XZ_	_—_

**Table 7:** Structure of chain III.

Chain	Basic Chain Structure	Chain Structure with Multi-Degree-of-Freedom Kinematic Pair	Chain Structure Containing Closed-Loop Structures
Chain III	U_r_P_X_P_Y_P_Z_R_Y_	U_r_P_X_P_Z_C_Y_	U_r_P_X_Pa_Y_C_Y_; U_r_Pa_X_Pa_Y_C_Y_; U_r_Pa_X_Pa_Y_Pa_Z_R_Y_
	U_r_P_Y_P_X_R_Y1_R_Y2_	U_r_P_X_R_Y_C_Y_	U_r_R_Y_Pa_Z_C_Y_; U_r_P_X/Y_Pa_Z_R_Y1_R_Y2_; U_r_Pa_Z_Pa_Z_R_Y1_R_Y2_
	U_r_P_Y_P_Z_R_Y1_R_Y2_	U_r_P_Z_R_Y_C_Y_	U_r_R_Y_Pa_X_C_Y_; U_r_P_Y_Pa_X_R_Y1_R_Y2_; U_r_Pa_X_Pa_X_R_Y1_R_Y2_
	U_r_P_Y_R_Y1_R_Y2_R_Y3_	U_r_R_Y1_R_Y2_C_Y_	U_r_Pa_X_R_Y1_R_Y2_R_Y3_

**Table 8:** Complexity of chain I.

Chain Type	Kinematic Pair Type	Complexity
Chain structure containing multi-degree-of-freedom kinematic pair	P_Z_S	1.7
	C_Z_U_XY_	1.95

**Table 9:** Complexity of chain II.

Chain Structure	Complexity	Chain Structure Containing Multi-Degree-of-Freedom Kinematic Pair	Complexity
R_Y_P_X_P_Y_P_Z_R_X_R_Z_	1.75	R_Y_P_X_P_Y_P_Z_U_XZ_; C_Y_P_X_P_Z_U_XZ_; C_Y_C_Z_C_X_	1.8 2.03 2.6
R_Y_P_Y_P_X_R_X1_R_X2_R_Z_	1.63	R_Y_P_X_P_Y_R_X_U_XZ_; R_Y_P_Y_C_X_U_XZ_; C_Y_C_X_U_XZ_	1.66 1.85 2.167
R_Y_P_Z_P_X_R_X1_R_X2_R_Z_	1.63	R_Y_P_Z_P_X_R_X_U_XZ_; R_Y_P_Z_C_X_U_XZ_; R_Y_R_X_C_Z_C_X_	1.66 1.85 2
R_Y_P_Y_P_Z_R_X_R_Z1_R_Z2_	1.63	R_Y_P_Y_P_Z_R_Z_U_XZ_; R_Y_P_Y_C_Z_U_XZ_; C_Y_C_Z_U_XZ_	1.66 1.85 2.167
R_Y_P_X_P_Z_R_X_R_Z1_R_Z2_	1.63	R_Y_P_X_P_Z_R_Z_U_XZ_; R_Y_P_X_C_Z_U_XZ_; R_Y_R_Z_C_X_C_Z_	1.66 1.85 2
R_Y_P_Y_R_X1_R_X2_R_Z1_R_Z2_	1.52	R_Y_P_Y_R_X_R_Z_U_XZ_; R_Y_P_Y_U_1XZ_U_2XZ_; C_Y_U_1XZ_U_2XZ_	1.52 1.525 1.733
R_Y_P_X_R_X1_R_X2_R_X3_R_Z_	1.52	R_Y_P_X_R_X1_R_X2_U_XZ_; R_Y_R_X_C_X_U_XZ_	1.52 1.675
R_Y_P_X_R_X1_R_X2_R_Z1_R_Z2_	1.52	R_Y_P_X_R_X_R_Z_U_XZ_; R_Y_P_X_U_1XZ_U_2XZ_; R_Y_R_Z_C_X_U_XZ_	1.52 1.525 1.675
R_Y_P_Z_R_X1_R_X2_R_Z1_R_Z2_	1.52	R_Y_P_Z_R_X_R_Z_U_XZ_; R_Y_P_Z_U_1XZ_U_2XZ_; R_Y_R_X_C_Z_U_XZ_	1.52 1.525 1.675
R_Y_P_Z_R_X_R_Z1_R_Z2_R_Z3_	1.52	R_Y_P_Z_R_Z1_R_Z2_U_XZ_; R_Y_C_Z_R_Z_U_XZ_	1.52 1.675
R_Y_R_X1_R_X2_R_Z1_R_Z2_R_Z3_	1.4	R_Y_R_X_R_Z1_R_Z2_U_XZ_; R_Y_R_Z_U_1XZ_U_2XZ_	1.38 1.35
R_Y_R_X1_R_X2_R_X3_R_Z1_R_Z2_	1.4	R_Y_R_Z_R_X1_R_X2_U_XZ_; R_Y_R_X_U_1XZ_U_2XZ_	1.38 1.35

**Table 10:** Complexity of chain II with closed-loop structure.

Chain II Structure Containing Closed-Loop Structures	Complexity
R_Y_P_X_P_Y_Pa_Y_U_XZ_	1.92
R_Y_P_X/Y_Pa_X/Y_Pa_X/Y_U_XZ_	2.04
R_Y_Pa_Y/X_Pa_X/Y_Pa_X/Y_U_XZ_	2.16
C_Y_P_X/Z_Pa_Y_U_XZ_	2.175
C_Y_Pa_Y_Pa_Y_U_XZ_	2.325
R_Y_P_X_Pa_Z_R_X_U_XZ_; R_Y_P_Z_Pa_Y_R_X_U_XZ_; R_Y_P_Y_Pa_X_R_Z_U_XZ_; R_Y_P_X_Pa_Y_R_Z_U_XZ_	1.78
R_Y_Pa_Z_Pa_Z_R_X_U_XZ_; R_Y_Pa_Y_Pa_Y_R_X_U_XZ_; R_Y_Pa_X_Pa_X_R_Z_U_XZ_; R_Y_Pa_Y_Pa_Y_R_Z_U_XZ_	1.9
R_Y_Pa_Z_C_X_U_XZ_; R_Y_Pa_Y_C_X_U_XZ_; R_Y_Pa_X_C_Z_U_XZ_; R_Y_Pa_Y_C_Z_U_XZ_	2
R_Y_Pa_X/Z_R_X_R_Z_U_XZ_; R_Y_Pa_Y/Z_R_X1_R_X2_U_XZ_; R_Y_Pa_Y/Z_R_X_R_Z_U_XZ_; R_Y_Pa_Y/X_R_Z1_R_Z2_U_XZ_; R_Y_Pa_Y/X_R_X_R_Z_U_XZ_	1.64
R_Y_Pa_X/Z_U_1XZ_U_2XZ_; R_Y_Pa_Y/Z_U_1XZ_U_2XZ_; R_Y_Pa_Y/X_U_1XZ_U_2XZ_	1.675

**Table 11:** Complexity of chain III.

Chain Structure	Complexity	Chain Structure Containing Multi-Degree-of-Freedom Kinematic Pair	Complexity	Chain Structure Containing a Closed-Loop Structure	Complexity
U_r_P_X_P_Y_P_Z_R_Y_	1.82	U_r_P_X_P_Z_C_Y_	2.05	U_r_P_X_Pa_Y_C_Y_; U_r_Pa_X_Pa_Y_C_Y_; U_r_Pa_X_Pa_Y_Pa_Z_R_Y_	2.2 2.35 2.18
U_r_P_Y_P_X_R_Y1_R_Y2_	1.68	U_r_P_X_R_Y_C_Y_	1.875	U_r_R_Y_Pa_Z_C_Y_; U_r_P_X/Y_Pa_Z_R_Y1_R_Y2_; U_r_Pa_Z_Pa_Z_R_Y1_R_Y2_	2.025 1.8 1.92
U_r_P_Y_P_Z_R_Y1_R_Y2_	1.68	U_r_P_Z_R_Y_C_Y_	1.875	U_r_R_Y_Pa_X_C_Y_; U_r_P_Y_Pa_X_R_Y1_R_Y2_; U_r_Pa_X_Pa_X_R_Y1_R_Y2_	2.025 1.8 1.92
U_r_P_Y_R_Y1_R_Y2_R_Y3_	1.54	U_r_R_Y1_R_Y2_C_Y_	1.7	U_r_Pa_X_R_Y1_R_Y2_R_Y3_	1.66

**Table 12:** D-H parameters of chain II.

*i*	α_i−1_/(°)	*a*_i−1_/mm	*θ*_*i*_/(°)	d_i_/mm
1	0	0	*θ*_2__1_	0
2	−90	*l*_21_	*θ*_22_ + 90	0
3	−90	0	*θ*_23_ + 90	0
4	0	*a*_24_	*θ*_24_	*l*_24_
5	−90	0	*θ*_2__5_	0
6	0	*l*_25_	*θ*_2__6_	0

**Table 13:** D-H parameters of chain III in mammalian model.

*i*	*α*_*i*−1_/(°)	*a*_*i*−1_/mm	*θ*_*i*_/(°)	*d*_*i*_/mm
1	0	0	0	*d*_31_
2	90	*l*_31_	*θ*_32_ − 90	0
3	−90	*l*_32_	*θ*_3__3_	0
4	0	*l*_33_	*θ*_3__4_	0
5	0	*l*_34_	*θ*_3__5_	0

**Table 14:** D-H parameters of chain III in insect model.

*i*	*α*_*i*−1_/(°)	*a*_*i*−1_/mm	*θ*_*i*_/(°)	*d*_*i*_/mm
1	0	0	0	*d*_31_
2	90	0	*θ*_32_	*l*_31_
3	−90	0	*θ*_3__3_	*l*_32_
4	0	*l*_33_	*θ*_34_ − 90	0
5	0	*l*_34_	*θ*_3__5_	0

**Table 15:** Constraint ranges of design parameters.

Design Variables	Constraint Range
*R* (mm)	[250, 450]
*l*_31_ (mm)	[50, 150]
*r* (mm)	[100, 220]

**Table 16:** Parameters of particle swarm optimization algorithm.

Parameter	Population Size	Number of Iterations	Social Learning Factor	Inertia Weight
Numerical value	50	80	2.0	0.99

**Table 17:** Optimization results of structural parameters.

Structural Parameters	*R*/mm	*l*_31_/mm	*r*/mm
Before optimization	300	90	160
After optimization	441.4536	143.5765	122.2757

**Table 18:** Performance index optimization results.

Mode	Performance Indicators	*W_V_*	*ζ*
Mammalian mode	Before optimization	0.4144	0.9332
	After optimization	0.7310	0.8870
Insect mode	Before optimization	0.5165	0.1981
	After optimization	0.5385	0.2059

## Data Availability

The original contributions presented in this study are included in the article. Further inquiries can be directed to the corresponding authors.

## References

[1] Ni L.W., Wu L., Zhang H.S. (2022). Parameters uncertainty analysis of posture control of a four-wheel-legged robot with series slow active suspension system. Mech. Mach. Theory.

[2] Grand C., Benamar F., Plumet F., Bidaud P. (2014). Stability and traction optimization of a reconfigurable wheel-legged robot. Int. J. Rob. Res..

[3] Grand C., Benamar F., Plumet F. (2010). Motion kinematics analysis of wheeled-legged rover over 3D surface with posture adaptation. Mech. Mach. Theory.

[4] Klemm V., Morra A., Salzmann C., Tschopp F., Bodie K., Gulich L., Kung N., Mannhart D., Pfister C., Vierneisel M. Ascento: A two-wheeled jumping robot. Proceedings of the 2019 International Conference on Robotics and Automation (ICRA).

[5] Tedeschi F., Carbone G. (2017). Design of a novel leg-wheel hexapod walking robot. Robotics.

[6] Hutter M., Gehring C., Lauber A., Gunther F., Bellicoso C.D., Tsounis V., Fankhauser P., Diethelm R., Bachmann S., Bloesch M. (2017). ANYmal-toward legged robots for harsh environments. Adv. Rob..

[7] Hutter M., Gehring C., Jud D., Lauber A., Bellicoso C.D., Tsounis V., Hwangbo J., Bodie K., Fankhauser P., Bloesch M. ANYmal a highly mobile and dynamic quadrupedal robot. Proceedings of the 2016 IEEE/RSJ International Conference on Intelligent Robots And Systems (IROS).

[8] Niu J.Y., Wang H.B., Shi H.M., Pop N., Li D., Li S.S., Wu S.Z. (2018). Study on structural modeling and kinematics analysis of a novel wheel-legged rescue robot. Int. J. Adv. Rob. Syst..

[9] Xu K., Ding X.L. (2013). Typical gait analysis of a six-legged robot in the context of metamorphic mechanism theory. Chin. J. Mech. Eng..

[10] Zeng D.X., Jing G.N., Su Y.L., Wang Y., Hou Y.L. (2016). A novel decoupled parallel mechanism with two translational and one rotational degree of freedom and its performance indices analysis. Adv. Mech. Eng..

[11] Cao Y., Chen H., Qin Y.L., Liu K., Ge S.Y., Zhu J.Y., Wang K., Yu J.H., Ji W.X., Zhou H. (2016). Type synthesis of fully-decoupled three-rotational and one-translational parallel mechanisms. Int. J. Adv. Rob. Syst..

[12] Xu Y.D., Wang B., Wang Z.F., Zhao Y., Liu W.L., Yao J.T., Zhao Y.S. (2019). Investigations on the principle of full decoupling and type synthesis of 2R1T and 2R parallel mechanisms. Trans. Can. Soc. Mech. Eng..

[13] Qu S.W., Li R.Q., Ma C.S., Li H. (2021). Type synthesis for lower-mobility decoupled parallel mechanism with redundant constraints. J. Mech. Sci. Technol..

[14] Zhang Y.B., Wei X.M., Zhang S., Chang Z.Z., Li Y.G. (2023). Type synthesis of the fully-decoupled two-rotational and one-translational parallel mechanism. J. Mech. Sci. Technol..

[15] Li S.H., Wang S., Li H.R., Wang Y.J., Chen S. (2023). Type synthesis of fully decoupled three translational parallel mechanism with closed-loop units and high stiffness. Chin. J. Mech. Eng..

[16] Wang S., Li S.H., Li H.R., Zhou Y.J., Wang Y.J., Wang X.Y. (2022). Type synthesis of 3T2R decoupled hybrid mechanisms with large bearing capacity. J. Mech. Sci. Technol..

[17] Wang R.Q., Song Y.Q., Dai J.S. (2021). Reconfigurability of the origami-inspired integrated 8R kinematotropic metamorphic mechanism and its evolved 6R and 4R mechanisms. Mech. Mach. Theory.

[18] Hu X.Y., Liu H.Z. (2022). Design and analysis of full-configuration decoupled actuating reconfigurable parallel spherical joint. J. Mech. Sci. Technol..

[19] Palpacelli M.C., Carbonari L., Palmieri G. (2016). Details on the design of a lockable spherical joint for robotic applications. J. Intell. Robot. Syst..

[20] Ye W., Chai X.X., Zhang K.T. (2020). Kinematic modeling and optimization of a new reconfigurable parallel mechanism. Mech. Mach. Theory.

[21] Yuan Y.T., Li D.L., Zhang D.H. Configuration change and kinematics analysis of a novel reconfigurable parallel mechanism with metamorphic joint. Proceedings of the 2024 6th International Conference on Reconfigurable Mechanisms and Robots.

[22] Kuang Y.L., Qu H.B., Li X., Wang X.L., Guo S. (2024). Design and singularity analysis of a parallel mechanism with origami-inspired reconfigurable 5R closed-loop linkages. Robotica.

[23] Zhong Y.H., Wang R.X., Feng H.S., Chen Y.S. (2019). Analysis and research of quadruped robot’s legs: A comprehensive review. Int. J. Adv. Rob. Syst..

[24] Zeng D.X., Hou Y.L., Lu W.J., Chang W., Huang Z. (2014). Type synthesis method for the translational decoupled parallel mechanism based on screw theory. J. Harbin Inst. Technol. (New Ser.).

[25] Ding H.F., Cao W.A., Cai C.W., Kecskeméthy A. (2015). Computer-aided structural synthesis of 5-DOF parallel mechanisms and the establishment of kinematic structure databases. Mech. Mach. Theory.

[26] Shen H.P., Tang Y., Wu G.L., Li J., Li T., Yang T.L. (2021). Design and analysis of a class of two-limb non-parasitic 2T1R parallel mechanism with decoupled motion and symbolic forward position solution-influence of optimal arrangement of limbs onto the kinematics, dynamics and stiffness. Mech. Mach. Theory.

[27] He J., Gao F., Meng X.D., Guo W.Z. (2015). Type synthesis for 4-DOF parallel press mechanism using G_F_ set theory. Chin. J. Mech. Eng..

[28] Caro S., Khan W.A., Pasini D., Angeles J. (2010). The rule-based conceptual design of the architecture of serial Schönflies-motion generators. Mech. Mach. Theory.

[29] Zhang J.Z., Jin Z.L., Feng H.B. (2018). Type synthesis of a 3-mixed-DOF protectable leg mechanism of a firefighting multi-legged robot based on G_F_ set theory. Mech. Mach. Theory.

[30] Wang J.S., Wu C., Liu X.J. (2010). Performance evaluation of parallel manipulators: Motion/force transmissibility and its index. Mech. Mach. Theory.

[31] Meng Q.Z., Xie F.G., Liu X.J. (2020). Motion-force interaction performance analyses of redundantly actuated and overconstrained parallel robots with closed-loop subchains. Mech. Mach. Theory.

[32] Mcghee R.B., Frank A.A. (1968). On stability properties of quadruped creeping gaits. Math. Biosci..

[33] de Santos P.G., Garcia E., Estremera J. (2007). Quadrupedal Locomotion: An Introduction to the Control of Four-legged Robots.

